# H3K36me3‐Guided m^6^A Modification of Oncogenic L1CAM‐AS1 Drives Macrophage Polarization and Immunotherapy Resistance in Hepatocellular Carcinoma

**DOI:** 10.1002/advs.202414909

**Published:** 2025-06-19

**Authors:** Teng Wang, Linyu Han, Yanfei Huo, Long Zhang, Yizhou Huang, Nasha Zhang, Ming Yang

**Affiliations:** ^1^ Shandong University Cancer Center Jinan Shandong Province 250100 China; ^2^ Shandong Provincial Key Laboratory of Precision Oncology Cancer Research Center Shandong Cancer Hospital and Institute Jinan Shandong Province 250117 China; ^3^ Department of Radiation Oncology Shandong Cancer Hospital and Institute Shandong First Medical University and Shandong Academy of Medical Sciences Jinan Shandong Province 250117 China; ^4^ Jiangsu Key Lab of Cancer Biomarkers Prevention and Treatment Collaborative Innovation Center for Cancer Personalized Medicine Nanjing Medical University Nanjing Jiangsu Province 211166 China; ^5^ School of Life Sciences Shandong First Medical University and Shandong Academy of Medical Sciences Taian Shandong Province 271016 China

**Keywords:** m^6^A, H3K36me3, L1CAM‐AS1, hepatocellular carcinoma, macrophage polarization, immunotherapy resistance

## Abstract

Hepatocellular carcinoma (HCC) is one of the most lethal malignancies and epigenetic modifiers play a key role in HCC progression. Histone H3 trimethylation at lysine‐36 (H3K36me3) determines deposition of mRNA de novo *N* ^6^‐methyladenosine (m^6^A) modification. However, it remains largely elusive how long noncoding RNAs (lncRNAs) are selected for proper m^6^A methylation. The current study provides evidence for *L1CAM‐AS1* as a novel H3K36me3‐guided, m^6^A‐modified lncRNA through integration of genome‐wide H3K36me3 profiles and transcriptome‐wide m^6^A profiles of HCC cells. The crucial m^6^A‐modification site in *L1CAM‐AS1* exon 3 is recognized by IGF2BP1, leading to increased lncRNA stability. Oncogenic *L1CAM‐AS1* shows higher expression in HCC tissues than in normal specimens, and its elevated expression is associated with shorten patient survival. Mechanistically, L1CAM‐AS1 interrupts binding of RAN to the E3 ligase OSTM1, suppresses RAN ubiquitination at Lys152 and Lys167, stabilizes RAN protein, enhances nuclear import of RELA (p65), and activates the NF‐κB signaling, leading to up‐regulated *CCL2* expression. L1CAM‐AS1‐induced CCL2 secretion from HCC cells enhances M2 polarization of tumor‐associated macrophages (TAMs). Meanwhile, immunosuppressive M2 macrophages‐released CCL5 augments RELA nuclear import in HCC cells, which in turn activates the NF‐κB signaling. Given the critical role of macrophages in anti‐tumor immunity, inhibition of the L1CAM‐AS1‐RAN axis promotes the efficacy of PD‐1 blockade via TAM reprogramming in HCC mouse models. In conclusion, this study provides novel insights into how epigenetic alternations are involved in antitumor immunity modulation and illustrates promising potentials of L1CAM‐AS1 in immune‐checkpoint inhibitor treatments for HCC.

## Graphical abstract

L1CAM‐AS1 is identified as a novel H3K36me3‐guided, m^6^A‐modified long noncoding RNA (lncRNA) in hepatocellular carcinoma (HCC) cells. L1CAM‐AS1 stabilizes RAN protein, enhances M2 macrophages‐released CCL5‐induced nuclear import of RELA, activates the NF‐κB signaling, up‐regulates *CCL2* expression and secretion from HCC cells, and enhances M2 polarization of macrophages. Inhibition of the L1CAM‐AS1‐RAN axis promotes the efficacy of PD‐1 blockade in HCC.

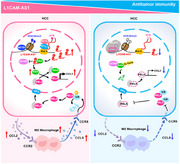

## 1 Introduction

As the most prevalent RNA modification, *N* ^6^‐Methyladenosine (m^6^A) is involved in control of RNA stability, splicing, export, and translation in mammalian cells.^[^ ^1^, ^2^, ^3^ ^]^ Only a portion of transcripts containing the RRACH (R = G or A; H = A, C or U) motif could be recognized and methylated by the METTL3‐METTL14 methyltransferase complex. Interestingly, histone H3 trimethylation at lysine‐36 (H3K36me3) modification determines which individual RNAs and specific RRACH motifs are deposited of de novo m^6^A RNA methylation.^[^ ^3^ ^]^ In line with this, knockdown of the histone methyltransferase SET domain containing 2 (SETD2), which catalyzing H3K36me2 to H3K36me3, suppressed cellular H3K36me3 and led to a significant decrease of m^6^A levels in total RNAs.^[^ ^3^ ^]^ Multiple protein coding genes, i.e. *MYC*, showed H3K36me3‐guided m^6^A modification across mRNAs.^[^ ^3^ ^]^ It remains elusive how long noncoding RNAs (lncRNAs) are selected for proper exposition of m^6^A methylation in cells.

Hepatocellular carcinoma (HCC) is one of the most prevalent neoplasms and the third lethal cancer globally, with a 5‐year survival rate of only 18%.^[^ ^4^ ^]^ The outcomes of advanced HCC patients who received systemic therapies containing immune‐checkpoint inhibitors (ICIs), havebeen significantly improved.^[^ ^5^, ^6^, ^7^, ^8^ ^]^ For instance, promising results of the IMbrave 050 trial showed survival benefits in HCC patients with a high relapse‐risk after surgical resection or local ablation who were treated with adjuvant atezolizumab (an anti‐PD‐L1 ICI) plus bevacizumab.^[^ ^9^ ^]^ This led to the approval of atezolizumab as the first‐line therapy for HCC. Similarly, the phase I/II CheckMate 040 trial indicated that Nivolumab, which was an anti‐PD1 monoclonal antibody (mAb), showed clinical improvements for HCC and, thus, was approved as a second line HCC treatment. However, not all HCC patients treated with ICIs achieved durable responses and evident improvements in survival. As a result, a systematical characterization of mechanisms on immunotherapy response and resistance would provide more efficient therapeutic approaches for HCC.

As the major components of HCC tumor microenvironment (TME), tumor‐associated macrophages (TAMs) can be polarized into two main subtypes (M1 and M2). M2 macrophages exert immunosuppressive functions.^[^ ^10^, ^11^ ^]^ Consistently, increased CD206^+^ M2 macrophage levels have been associated with aggressive phenotypes of HCC patients, such as advanced disease stages, poor overall survival (OS), and shortened time to recurrence.^[^ ^11^ ^]^ CCL2 is an important TAM chemoattractant and blockade of the CCL2‐CCR2 axis inhibited monocyte/TAM recruitment and M2 polarization, which thus activates the antitumor response of CD8^+^ T cells in HCC.^[^ ^12^ ^]^ A phase II clinical trial (NCT04123379) is currently testing Nivolumab in combination with a CCR2/5‐inhibitor. However, specific mechanisms underlying transcriptional regulation of *CCL2* overexpression in HCC, especially through the NF‐κB pathway, are largely unclear.

In the current study, we integrated transcriptome‐wide m^6^A methylation profiles and genome‐wide H3K36me3 modification profiles of HCC cells. As a result, we identified *L1CAM‐AS1* as a novel H3K36me3‐guided, m^6^A‐modified lncRNA in HCC. *L1CAM‐AS1* shows higher expression in HCC tissues than in normal specimens, and its high expression is associated with worse survival of HCC patients. L1CAM‐AS1 could facilitate proliferation, migration, and invasion of HCC cells in vitro and in vivo. L1CAM‐AS1 stabilizes RAN protein, activates the NF‐κB signaling and up‐regulates *CCL2* expression via promoting nuclear import of transcription factor (TF) RELA (p65). Importantly, L1CAM‐AS1 induces significantly increased CCL2 secretion from HCC cells and M2 polarization of macrophages, thereby inhibiting antitumor responses. On the other hand, immunosuppressive M2 TAMs‐secreted CCL5 augments RELA nuclear import, which in turn activates NF‐κB in HCC cells. Consistently, inhibition of the L1CAM‐AS1‐RAN axis promotes the efficacy of PD‐1 blockade via TAM reprogramming in HCC mouse models. Therefore, our study provides novel insights into the role of epigenetic alternations on antitumor immunity modulation, illustrating its promising potential for enhanced PD‐1 blockade therapy in HCC.

## 2 Results

### 2.1 L1CAM‐AS1 Is a Novel lncRNA with m^6^A‐Modification Guided by H3K36me3 in HCC

**Figure 1:**
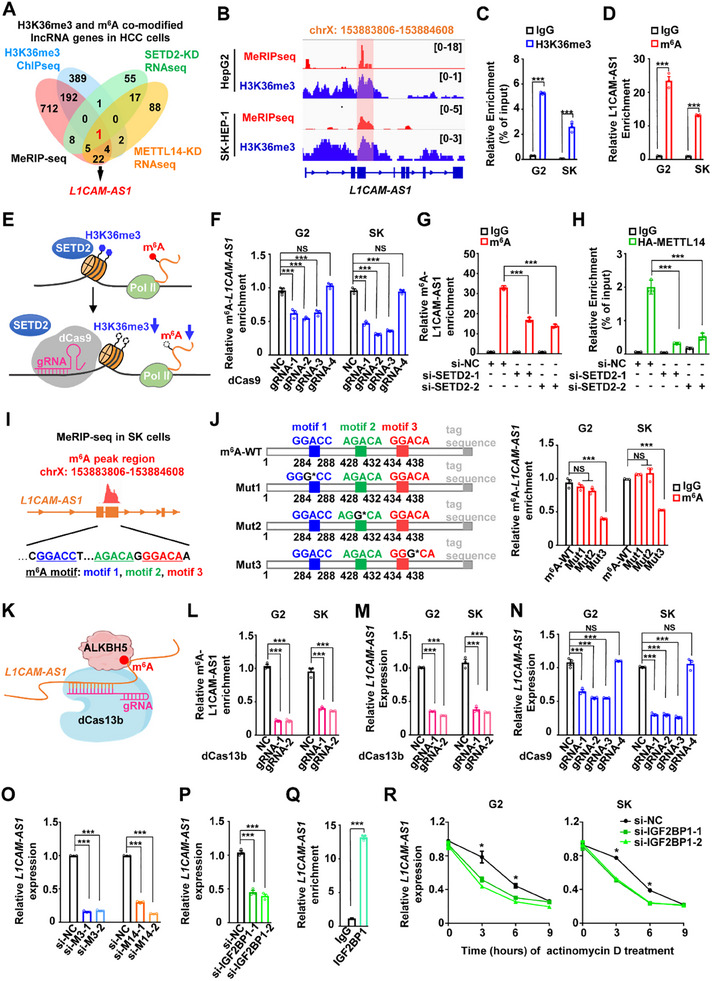
H3K36me3‐guided m^6^A modification of lncRNA L1CAM‐AS1 in hepatocellular carcinoma (HCC). A) A flowchart of genome‐wide identification H3K36me3 and m^6^A co‐modified lncRNA genes in HCC. B) The MeRIP‐seq and H3K36me3 ChIP‐seq signals around *L1CAM‐AS1*. The red peaks represent the MeRIP‐seq signal of the HepG2 cells (G2) and SK‐HEP‐1 cells (SK). The blue peaks represent H3K36me3 signals of G2 and SK HCC cell lines. C) The ChIP assays showed the H3K36me3 modification levels of *L1CAM‐AS1* in G2 and SK cells. IgG served as the control. D) The MeRIP assays showed the m^6^A modification levels of lncRNA L1CAM‐AS1 in G2 and SK cells. IgG served as the control. E) The diagram showed how the CRISPR/dCas9‐KRAB (CRISPRi) system inhibits H3K36me3 and m^6^A modifications in cells. F) The m^6^A modified‐L1CAM‐AS1 levels in HCC cells transfected with CRISPRi and different gRNAs. G) The MeRIP assays showed the m^6^A modification levels of L1CAM‐AS1 in the *SETD2*‐KD G2 cells. IgG served as the control. H) The RIP assays showed the association of METTL14 with L1CAM‐AS1 in the *SETD2*‐KD G2 cells. IgG served as the control. I) The MeRIP‐seq data showed the m^6^A peak in *L1CAM‐AS1* exon 3 in SK cells and three potential m^6^A‐modified sites (286A, 430A, and 436A) in this RNA region. J) Schematic diagram of human full‐length *L1CAM‐AS1* with a tag sequence and its three mutated forms used in m^6^A RIP assays. The MeRIP assays showed that the L1CAM‐AS1 436A is its m^6^A site in HCC cells. K) Schematic diagram of the m^6^A CRISPR/dCas13b‐ALKBH5 (dCas13b‐CRISPRi) system. L) The MeRIP assays showed the m^6^A modification levels of L1CAM‐AS1 in HCC cells transfected with the dCas13b‐CRISPRi system with two gRNAs targeting the lncRNA m^6^A site. M,N) The L1CAM‐AS1 expression in HCC cells transfected with the CRISPRi system or the dCas13b‐CRISPRi system. O,P) The L1CAM‐AS1 expression in HCC cells transfected with the siRNAs for *METTL3*, *METTL14* or *IGF2BP1*. Q) The RIP assays showed the association of m^6^A reader IGF2BP1 with L1CAM‐AS1 in G2 cells. IgG served as the control. R) Knockdown of *IGF2BP1* significantly decreased *L1CAM‐AS1* RNA stability in cells. Data information: Each value represents mean ± SD. The difference between two groups was calculated using Student's *t* test. One‐way ANOVA analysis with Dunnett's test was used for multiple comparisons. *p *<* 0.05; **p *<* 0.01; ***p *<* 0.001, NS, not significant. Data show one representative of three independent experiments with three biological replicates.

H3K36me3 modification directs deposition of de novo m^6^A methylation of various mRNAs.^[^ ^3^ ^]^ The methyltransferase METTL14, a core component of the m^6^A writer complex (METTL3/14‐WTAP) can directly bind to H3K36me3 which catalyzed by SETD2.^[^ ^3^ ^]^ This interaction facilitates the deposition of m^6^A modifications on nascent RNA during transcription elongation.^[^ ^3^ ^]^ However, it remains unclear how lncRNAs are selected for proper m^6^A methylation in human cells. To identify m^6^A‐modified lncRNAs which is guided by H3K36me3 in HCC (**Figure** **1**A), we conducted integration analyses using H3K36me3 ChIP‐seq data of HepG2, MeRIP‐seq data of HepG2, RNA‐seq data of HepG2 with or without *SETD2* silencing, and RNA‐seq data of HepG2 with or without *METTL14* silencing. This resulted in identification of one lncRNA gene, *L1CAM‐AS1* with H3K36me3 and m^6^A co‐modifications in HCC cells (Figure 1B). *L1CAM‐AS1* locates on chromosome Xq28 and is antisense lncRNA gene of *L1CAM*. This transcript is 1,392nt (NR_130768.1). The H3K36me3 and m^6^A co‐modifications of *L1CAM‐AS1* was further verified by H3K36me3 ChIP‐qPCR and MeRIP‐qPCR assays in HepG2 and SK‐Hep‐1 cells (Figure 1C,D).

To confirm whether m^6^A modification of L1CAM‐AS1 is H3K36me3‐driven, we suppressed H3K36me3 of *L1CAM‐AS1* locus using the dCas9‐CRISPRi system with four gRNAs (Figure 1E and Figure S1A, Supporting Information). Downregulation of *L1CAM‐AS1* m^6^A levels was observed in HCC cells transfected with gRNA‐1, gRNA‐2, and gRNA‐3 (all *p* < 0.001) (Figure 1F). Additionally, after silencing the H3K36me3 methyltransferase *SETD2* in HCC cells, we found significant reductions in *L1CAM‐AS1* m^6^A levels as well as METTL3/METTL14 binding to the lncRNA (all *p* < 0.001) (Figure 1G,H and Figure S1B–E, Supporting Information). These data demonstrate that *L1CAM‐AS1* is a novel lncRNA with m^6^A modification which is H3K36me3‐guided in HCC.

### 2.2 Stabilization of L1CAM‐AS1 by the m^6^A Modification and Its Reader Protein IGF2BP1

We then explored the m^6^A modification site(s) in *L1CAM‐AS1* and how m^6^A methylation affects the lncRNA expression. As shown in Figure 1I, there was an m^6^A peak within *L1CAM‐AS1* exon 3. Three potential m^6^A sites (286A, 430A and 436A) in *L1CAM‐AS1* exon 3 RNA were identified Using SK‐Hep‐1 MeRIP‐seq data and the SRAMP algorithm (http://www.cuilab.cn/sramp) (Figure 1J). Subsequent m^6^A‐specific RIP‐qPCR assays showed that cells with ectopic expression of *L1CAM‐AS1* mutant 3 had significantly lower m^6^A levels of *L1CAM‐AS1* compared to cells with ectopic m^6^A‐WT *L1CAM‐AS1* expression (both *p* < 0.001) (Figure 1J), suggesting that *L1CAM‐AS1* 436A is the lncRNA m^6^A site in HCC.

Multiple lines of evidence elucidated that RNA stability is regulated by m^6^A modification. To investigate this possibility, we suppressed *L1CAM‐AS1* m^6^A levels using the dCas13b‐CRISPRi system with two gRNAs targeting the lncRNA m^6^A site (Figure 1K,L). Downregulated *L1CAM‐AS1* expression was observed in cells transfected with gRNA‐1 and gRNA‐2 (both *p* < 0.001) (Figure 1M). Consistently, the dCas9‐CRISPRi gRNA‐1, gRNA‐2, and gRNA‐3 also evidently suppressed *L1CAM‐AS1* expression (all *p* < 0.001) (Figure 1N). Consistent with these findings, silencing of *SETD2* also suppressed *L1CAM‐AS1* expression in HCC cells (Figure S1K, Supporting Information). In support of this, knock‐down of *METTL3* or *METTL14*, two key m^6^A methyltransferases, reduced *L1CAM‐AS1* expression in HCC cells (all *p* < 0.001) (Figure 1O and Figure S1E–H, Supporting Information). Moreover, the RIP assays verified the interactions between the *L1CAM‐AS1* RNA and METTL3 or METTL14 protein (all *p* < 0.001) (Figure S1I,J, Supporting Information). These results indicate that m^6^A modification in *L1CAM‐AS1* stabilized the lncRNA.

IGF2BPs (IGF2BP1/2/3) are m^6^A readers stabilizing target RNAs in an m^6^A‐dependent way.^[^ ^13^ ^]^ Since the m^6^A modification of *L1CAM‐AS1* is essential for its RNA stabilization, we examined whether IGF2BPs act as m^6^A readers of L1CAM‐AS1 in HCC cells. Silencing *IGF2BP1*, but not *IGF2BP2* or *IGF2BP3*, significantly downregulated endogenous *L1CAM‐AS1* RNA levels (all *p* < 0.001) (Figure 1P and Figure S1K–Q, Supporting Information). Consistently, IGF2BP1 protein showed high binding affinity with the *L1CAM‐AS1* RNA in HCC cells (Figure 1Q and Figure S1R, Supporting Information). Importantly, knockdown of *IGF2BP1* markedly reduced the RNA stability of *L1CAM‐AS1* (Figure 1R). These data elucidated that IGF2BP1 is the m^6^A reader stabilizing *L1CAM‐AS1* in HCC.

### 2.3 L1CAM‐AS1 Is a Novel Oncogene in HCC

**Figure 2:**
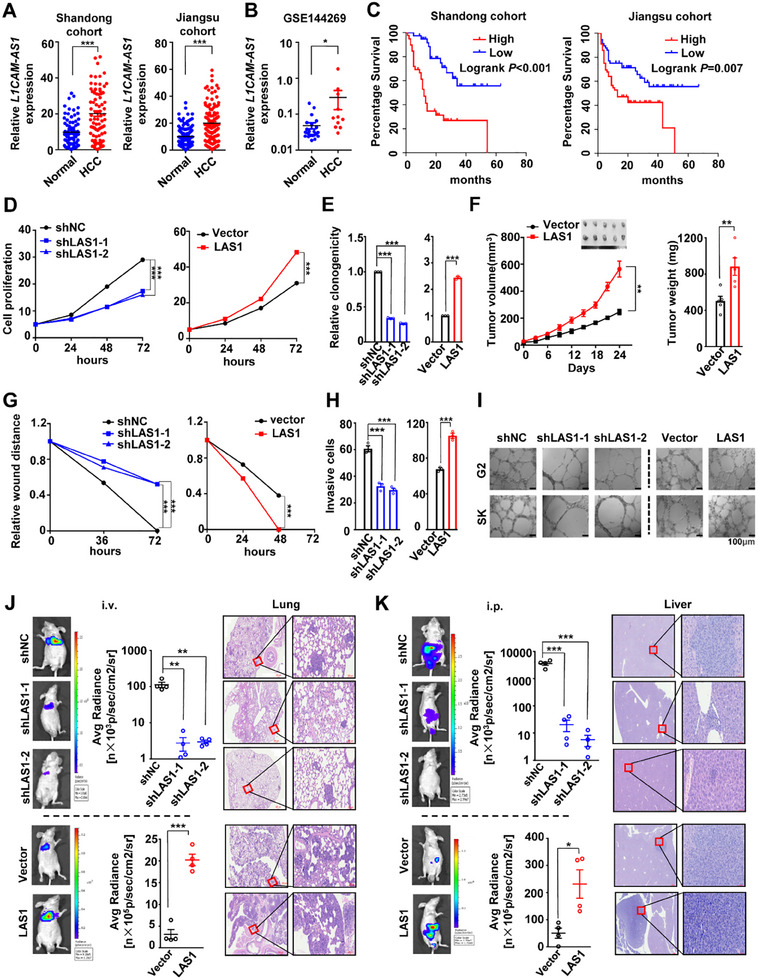
*L1CAM‐AS1* promotes proliferation, invasion and metastasis of HCC cells in vitro and in vivo. A,B) The *L1CAM‐AS1* expression levels in Shandong cohort, Jiangsu cohort, and the GSE144269 cohort. C) Kaplan–Meier plots of HCC patients’ overall survival time were stratified according to *L1CAM‐AS1* expression levels in Shandong cohort or Jiangsu cohort. D,E) *L1CAM‐AS1* significantly enhanced proliferation and clonogenicity of G2 cells. F) *L1CAM‐AS1* promoted the growth of HCC xenografts compared with control xenografts. G,H) *L1CAM‐AS1* significantly accelerated wound‐healing and invasion in G2 cells. I) *L1CAM‐AS1* promoted tube formation of HUVEC cells. J) Reduced or increased tumor metastasis was observed in the lungs of nude mice following tail vein injection (i.v.) of *L1CAM‐AS1*‐KD or *L1CAM‐AS1*‐OE SK cells (*n* = 4). Left panel: Luciferase activities of cancer cells were detected at the 40th day (shNC, shLAS1‐1, and shLAS1‐2) or the 35th day after injection (Vector and LAS1). Right panel: Representative images of hematoxylin and eosin‐stained (HE) slides of lung metastases. K) There was decreased or elevated tumor metastasis in the livers of nude mice following intraperitoneal injection (i.p.) of *L1CAM‐AS1*‐KD or *L1CAM‐AS1*‐OE SK cells (*n* = 4). Left panel: Luciferase activities of cancer cells were detected at the 35th day (shNC, shLAS1‐1, and shLAS1‐2) or the 30th day after injection (Vector and LAS1). Right panel: Representative images of hematoxylin and eosin‐stained (HE) slides of liver metastases. Data information: Each value represents mean ± SD. The difference between two groups was calculated using Student's *t* test. *p *<* 0.05; **p *<* 0.01; ***p *<* 0.001. Data show one representative of three independent experiments with three biological replicates.

Due to the role of *L1CAM‐AS1* in the pathogenesis of HCC remains elusive, we firstly detected its expression in tissue specimens from independent HCC patient cohorts (**Figure** **2**A,B). Our findings showed that there was significant increase of *L1CAM‐AS1* levels in HCC tissues compared to normal liver specimens in both Shandong cohort and Jiangsu cohort (both *p* < 0.001) (Figure 2A). In line with this, elevated *L1CAM‐AS1* levels were identified in malignant specimens versus normal tissues in a Mongolia HCC cohort (GSE144269) (*p* < 0.05) (Figure 2B). High *L1CAM‐AS1* expression in cancer specimens was correlated with a shortened OS time in Shandong and Jiangsu cohorts (both log‐rank *p* < 0.01) (Figure 2C). These data demonstrated that *L1CAM‐AS1* may act as a novel oncogene in HCC.

We next developed stable *L1CAM‐AS1*‐KD HCC cells (shNC, shLAS1‐1, and shLAS1‐2) and stable *L1CAM‐AS1*‐OE HCC cells (Vector and LAS1) (Figure S2A, Supporting Information). *L1CAM‐AS1* significantly promoted proliferation and colony formation of HepG2 and SK‐HEP‐1 cells (all *p* < 0.001) (Figure 2D,E and Figure S2B–D, Supporting Information). We also assessed the in vivo biological significance of *L1CAM‐AS1* (Figure 2F). Importantly, there were evidently enhanced proliferation and elevated tumor weights of *L1CAM‐AS1*‐OE xenografts compared to controls (*p* < 0.01) (Figure 2F), indicating the oncogenic role of *L1CAM‐AS1* in HCC.

We also investigated the role of *L1CAM‐AS1* during migration, invasion, and metastasis of HCC cells. *L1CAM‐AS1* promoted migration and invasion of HCC cells (Figure 2G,H and Figure S2E–I, Supporting Information). Additionally, we performed the tube formation assays using human HUVECs cultured with HCC cell conditioned medium (Figure 2I). Compared to the controls, the conditioned medium from *L1CAM‐AS1*‐KD HCC cells evidently reduced tube formation of HUVECs (Figure 2I). In contrast, tube formation was enhanced in HUVECs cultured with the medium from *L1CAM‐AS1*‐OE HCC cells (Figure 2I). We then explored if *L1CAM‐AS1* may impact in vivo metastasis of HCC cells through injecting HCC cells into mice via the tail vein or into the mouse abdomen. These models showed that stabilized *L1CAM‐AS1* depletion remarkably inhibited hematogenous metastasis or abdominal metastases of HCC cells (both *p* *< *0.001) (Figure 2J,K and Figure S3A,B, Supporting Information). On the contrary, *L1CAM‐AS1* over‐expression led to enhanced distant HCC metastases (*p* *< *0.001) (Figure 2J,K and Figure S3C,D, Supporting Information). The HE staining analyses of the metastasis tumors of lungs or livers from these mice further validated these results (Figure 2J,K). Furthermore, we constructed an orthotopic HCC model using the stably *Ran*‐KD Hepa1‐6 cells with overexpressed luciferase gene as well the control cells via orthotopic liver injection into C57BL/6 mice in the revised manuscript. Our results demonstrated that knocking‐down of *Ran* suppressed pulmonary metastases of orthotopic mouse tumors (*p* *<* 0.05) (Figure S3E, Supporting Information). Taken together, these findings provide compelling evidences that that L1CAM‐AS1 enhances HCC metastasis in vitro and in vivo.

### 2.4 Identification of RAN as the Interacting Protein of L1CAM‐AS1

**Figure 3:**
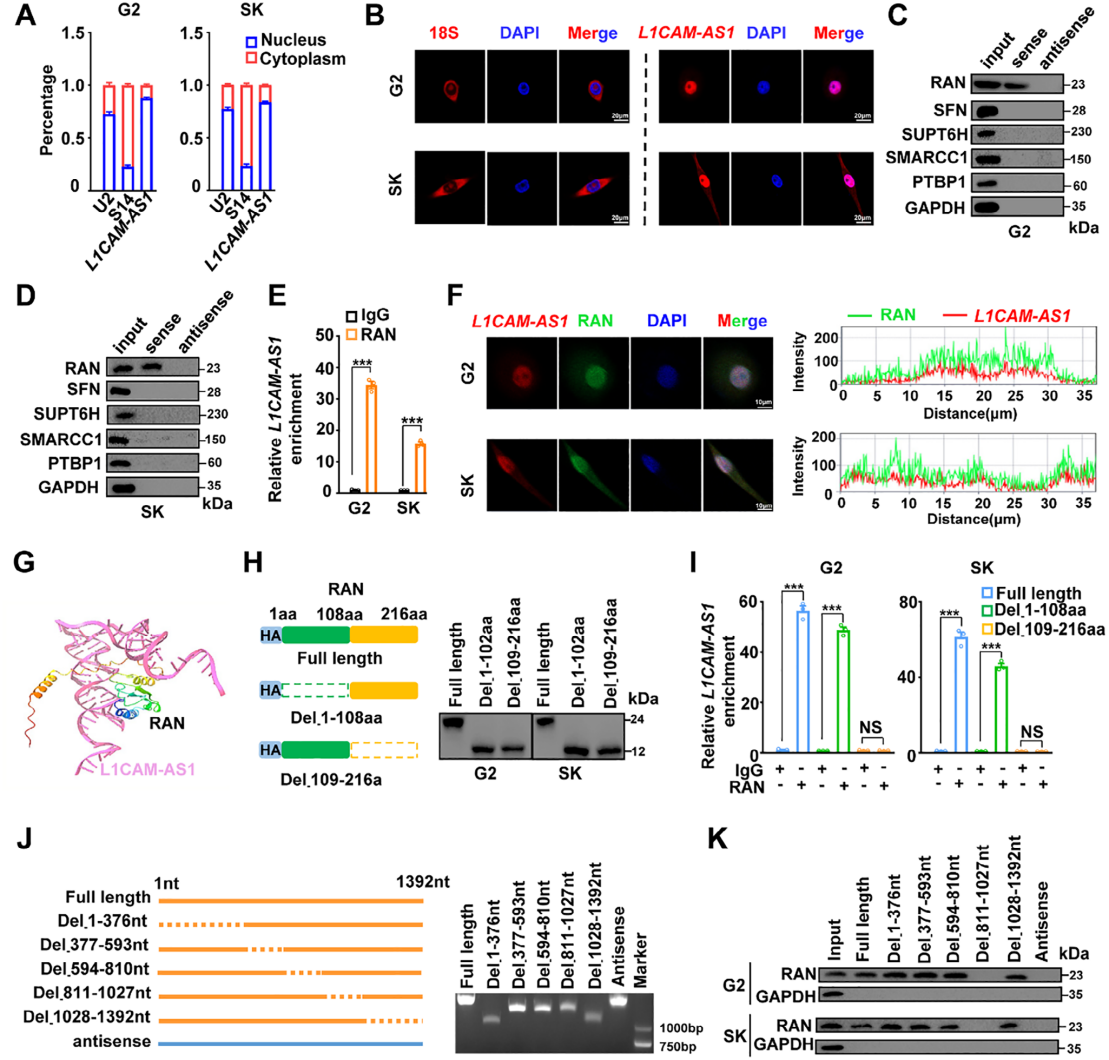
L1CAM‐AS1 interacts with RAN in HCC. A) Cellular location of L1CAM‐AS1 in G2 and SK cells. B) The FISH assays showed the cellular localization of L1CAM‐AS1 in G2 and SK cells. 18sRNA represents RNAs in the cytosolic fraction. C,D) L1CAM‐AS1 pulldown followed by Western blot validated its interaction with RAN and other candidate proteins identified by Mass spectrometry in HCC cells. E) The RIP assays showed an association of RAN with lncRNA L1CAM‐AS1 in G2 and SK cells. Relative enrichment (means ± SD) represents RNA levels associated with RAN relative to an input control from three independent experiments. IgG served as the control. F) The immunofluorescence and FISH assays showed co‐localization of L1CAM‐AS1 and RAN protein in HCC cells. Nuclei (blue) were stained with 4,6‐diamidino‐2‐phenylindole (DAPI). G) The interaction between L1CAM‐AS1 and RAN was predicted using HDOCK. H) Schematic representation of HA‐tagged RAN and its two truncated variants used in RIP assays. Western blot analyses verified full‐length and truncated forms of RAN protein. I) The RIP assays showed an association between 109aa‐216aa of RAN with L1CAM‐AS1 in G2 and SK cells. J) Schematic diagram of full‐length L1CAM‐AS1 and its truncated forms used in L1CAM‐AS1 pulldown assays. The full‐length and truncated forms of L1CAM‐AS1 were verified by agarose electrophoresis. K) Western blot analyses of RAN retrieved by in vitro transcribed biotinylated full‐length L1CAM‐AS1 or truncated forms of L1CAM‐AS1. Data information: Each value represents mean ± SD. The difference between two groups was calculated using Student's *t* test. One‐way ANOVA analysis with Dunnett's test was used for multiple comparisons. *p *<* 0.05; **p *<* 0.01; ***p *<* 0.001, NS, not significant. Data show one representative of three independent experiments with three biological replicates.

To elucidate the mechanisms underlying L1CAM‐AS1‐mediated HCC progression, we firstly detected where L1CAM‐AS1 is located in HCC cells and found that it is predominantly located in nucleus (**Figure** **3**A,B). Since lncRNAs can interact with different proteins in cells, we performed RNA pulldown plus mass spectrometry proteomics to identify L1CAM‐AS1‐binding protein(s) using SK‐Hep‐1 cellular extracts (Table S4, Supporting Information). Independent RNA pulldown assays successfully verified RAN, a small GTP binding protein, as the lncRNA‐binding protein in HCC cells (Figure 3C,D). Consistently, Gene Ontology (GO) analyses of differentially expressed genes in *L1CAM‐AS1*‐KD cells showed significant enrichments of genes involved in the GTPase regulatory pathways (Figure S4A, Supporting Information). RIP assays showed significant enrichments of L1CAM‐AS1 in the RNA‐RAN complexes in HCC cells (both *p* *<* 0.001) (Figure 3E). According to RNA FISH and immunofluorescence assays, L1CAM‐AS1 and RAN colocalized in HCC cell nucleus (Figure 3F). In support of this, the RNA–protein interaction was further confirmed using docking analyses through the HDOCK algorithm (http://hdock.phys.hust.edu.cn) (Figure 3G). Collectively, these data demonstrated that RAN protein interacts with L1CAM‐AS1 RNA.

The RIP assays demonstrated that the 109aa‐216aa region of RAN protein was essential for its interaction with L1CAM‐AS1 RNA (Figure 3H,I). In order to examine the specific RNA regions needed for the RNA–protein interaction, we constructed multiple truncated *L1CAM‐AS1* plasmids (Figure 3J and Figure S4B, Supporting Information). The nucleotides 811–1027 RNA region of L1CAM‐AS1 is required for the RNA–protein interaction, as demonstrated by the RNA pulldown assays (Figure 3K). Moreover, silencing of *RAN* with siRNAs significantly inhibited the oncogenic proliferation, migration, and invasion of the *L1CAM‐AS1*‐OE HCC cells (Figure S4C–I).

### 2.5 L1CAM‐AS1 Stabilizes RAN Protein via Blocking RAN Ubiquitination at Lys152 and Lys167 by the E3 Ligase OSTM1

**Figure 4:**
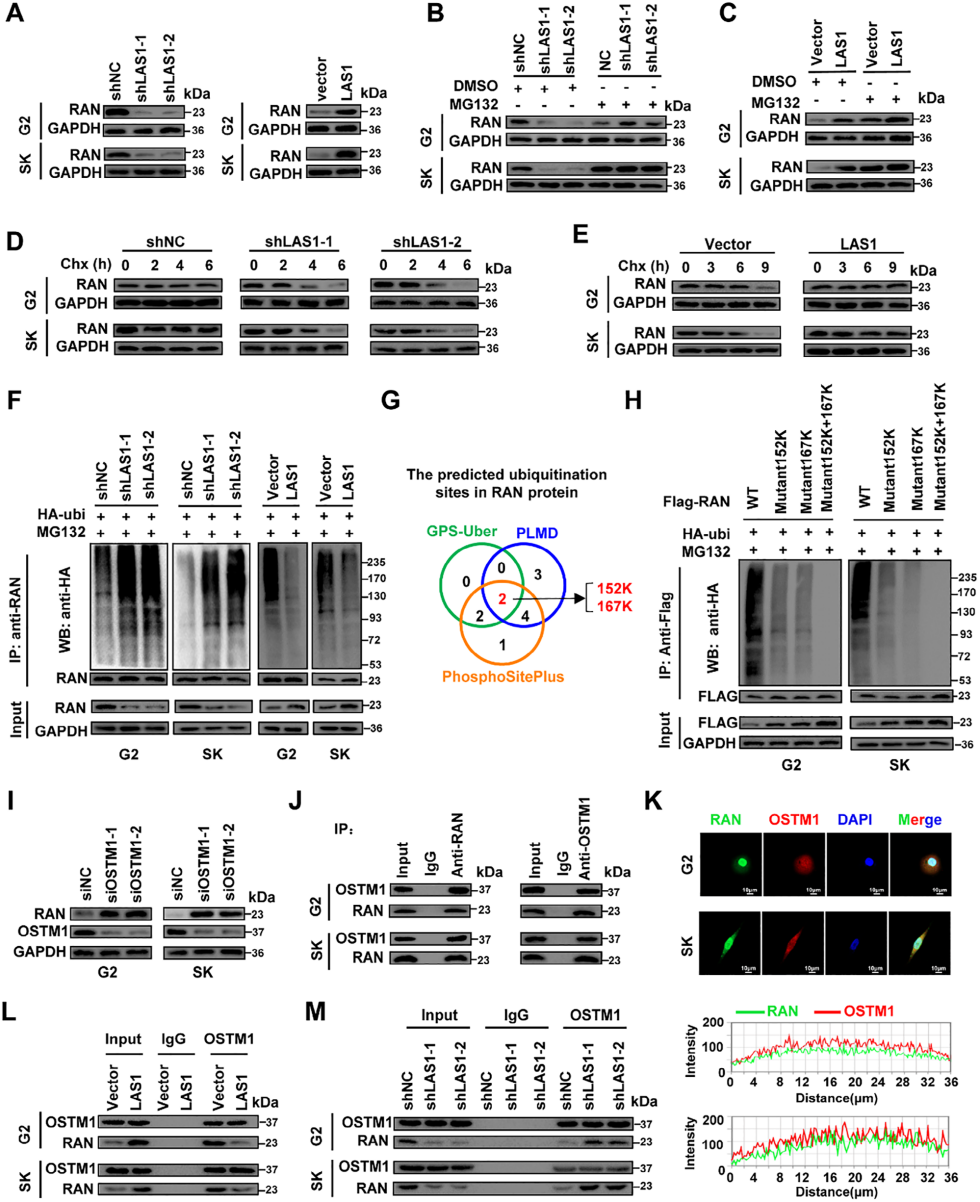
L1CAM‐AS1 stabilizes RAN through interrupting the interaction between RAN and the E3 ligase OSTM1. A) Western blot analyses of RAN protein in *L1CAM‐AS1*‐KD or *L1CAM‐AS1*‐OE HCC cells. B,C) MG132 treatment eliminated L1CAM‐AS1‐induced RAN protein up‐regulation in cells. D,E) RAN protein levels in *L1CAM‐AS1*‐KD or *L1CAM‐AS1*‐OE HCC cells with cycloheximide (CHX) for the indicated periods of time. F) Western blot of the ubiquitination of RAN protein in *L1CAM‐AS1*‐KD or *L1CAM‐AS1*‐OE cells. G) Prediction of the RAN ubiquitination sites (K152 and K167) via three bioinformatics algorithms (GPS‐Uber, PLMD, and PhosphoSitePlus). H) RAN ubiquitination assays indicated that 152K and 167K are key ubiquitination sites of RAN in HCC cells. I) Knocking‐down of the E3 ligase OSTM1 expression elevated RAN protein levels in HCC cells. J) Interactions between RAN and OSTM1 were verified via Co‐IP assays in cells. K) Immunofluorescence staining results showed co‐localization of OSTM1 and RAN proteins in HCC cells. Nuclei (blue) were stained with DAPI. L,M) L1CAM‐AS1 diminished interactions between RAN and OSTM1 in cells. Data information: si‐NC: negative control RNA; siOSTM1‐1: siRNA‐1 of *OSTM1*; siOSTM1‐2: siRNA‐2 of *OSTM1*. Data show one representative of three independent experiments with three biological replicates.

We next explored the molecular consequences of the L1CAM‐AS1–RAN interaction in HCC cells. Silencing of *L1CAM‐AS1* markedly down‐regulated RAN protein levels in HCC cells, whereas overexpressed *L1CAM‐AS1* elevated RAN protein (**Figure** **4**A). L1CAM‐AS1 did not disturb *RAN* mRNA levels in HCC cells (Figure S5A, Supporting Information). When MG132, a 26S protostome inhibitor, was applied to *L1CAM‐AS1*‐KD HCC cells, decreased RAN protein expression was observed (Figure 4B). On the other hand, *L1CAM‐AS1*‐induced up‐regulation of RAN protein in HCC cells was eliminated by MG132 (Figure 4C), suggesting that the lncRNA might control the proteasome degradation of RAN. To validate this, we measured RAN levels in HCC cells treated with the protein synthesis inhibitor CHX. It has been showed that the RAN protein levels declined much faster in the stable *L1CAM‐AS1*‐KD HCC cells in comparison with the control cells (Figure 4D). In contrast, the *L1CAM‐AS1*‐OE cells treated with CHX showed an obviously longer half‐life of RAN protein than in that in control cells (Figure 4E).

We then examined whether RAN ubiquitination facilitated RAN degradation under the control of lncRNA L1CAM‐AS1. There was a noticeable increase in the ubiquitination levels of RAN protein in the *L1CAM‐AS1*‐KD cells compared to the controls (Figure 4F). In line with this, the ubiquitination of RAN was decreased in the *L1CAM‐AS1*‐OE cells (Figure 4F). To explore the precise ubiquitination site(s) modulating RAN degradation, we identified two putative sites (Lys152 and Lys167, K152 and K167) via integration of three bioinformatics algorithms (GPS‐Uber, PLMD, and PhosphoSitePlus) (Figure 4G). We then successfully validated the importance of K152 and K167 in cells ectopic expression of the wide‐type RAN protein, the Mutant152K RAN protein, the Mutant167K RAN protein, or the double mutated RAN protein (Mutant152K+Mutant167K) (Figure S5B, Supporting Information). Mutant152K or Mutant167K alone remarkably suppressed RAN ubiquitination levels (Figure 4H). Interestingly, the double mutated RAN protein showed more evidently attenuated ubiquitination of RAN compared to Mutant152K or Mutant167K (Figure 4H). Notably, Lys152 and Lys167 locate in the RAN protein region (109aa‐216aa) essential for its interaction with L1CAM‐AS1 (Figure 3H,I). Taken together, these results elucidated that L1CAM‐AS1 stabilizes RAN protein by inhibiting its ubiquitination of Lys152 and Lys167 and proteasome degradation.

We then disclose how L1CAM‐AS1 suppresses the proteasome degradation of RAN. Two candidate E3 ligases (OSTM1 and FBXO32) were identified after systematical evaluation of proteins precipitated by RAN in HepG2 cells via mass spectrometry (Table S5, Supporting Information). To validate whether OSTM1 or FBXO32 is the E3 ligase of RAN, we initially measured RAN levels in cells after knocking‐down of *OSTM1* or *FBXO32* (Figure 4I and Figure S5C–E, Supporting Information). Silencing of *OSTM1* up‐regulated RAN protein expression in HCC cells in comparison with the controls (Figure 4I). Nevertheless, there were no such expression alterations after knocking‐down *FBXO32* expression in HCC cells (Figure S5D,E, Supporting Information). Importantly, endogenous OSTM1 or RAN can be immunoprecipitated with each other in HCC cells (Figure 4J). Co‐localization of OSTM1 and RAN in HCC cells was revealed by immunofluorescence assays (Figure 4K), demonstrating that OSTM1 is the putative E3 ligase of RAN. We next investigated whether L1CAM‐AS1 influences the binding of RAN with OSTM1. Less OSTM1 protein was precipitated with RAN in the stably *L1CAM‐AS1*‐OE cells compared to the control cells (Figure 4L). In contrast, more RAN protein could be precipitated with OSTM1 compared with the controls in the *L1CAM‐AS1*‐KD cells (Figure 4M). Furthermore, we found that OSTM1 specifically interacts with the 109‐216aa region of RAN, which is the same region interacting with L1CAM‐AS1 in HCC cells (Figure S5F, Supporting Information). These findings elucidate that L1CAM‐AS1 attenuates interactions between RAN and the E3 ligase OSTM1 and, thus, promotes RAN stabilization.

### 2.6 The L1CAM‐AS1‐RAN Axis Promoted M2 Polarization of Macrophages

**Figure 5:**
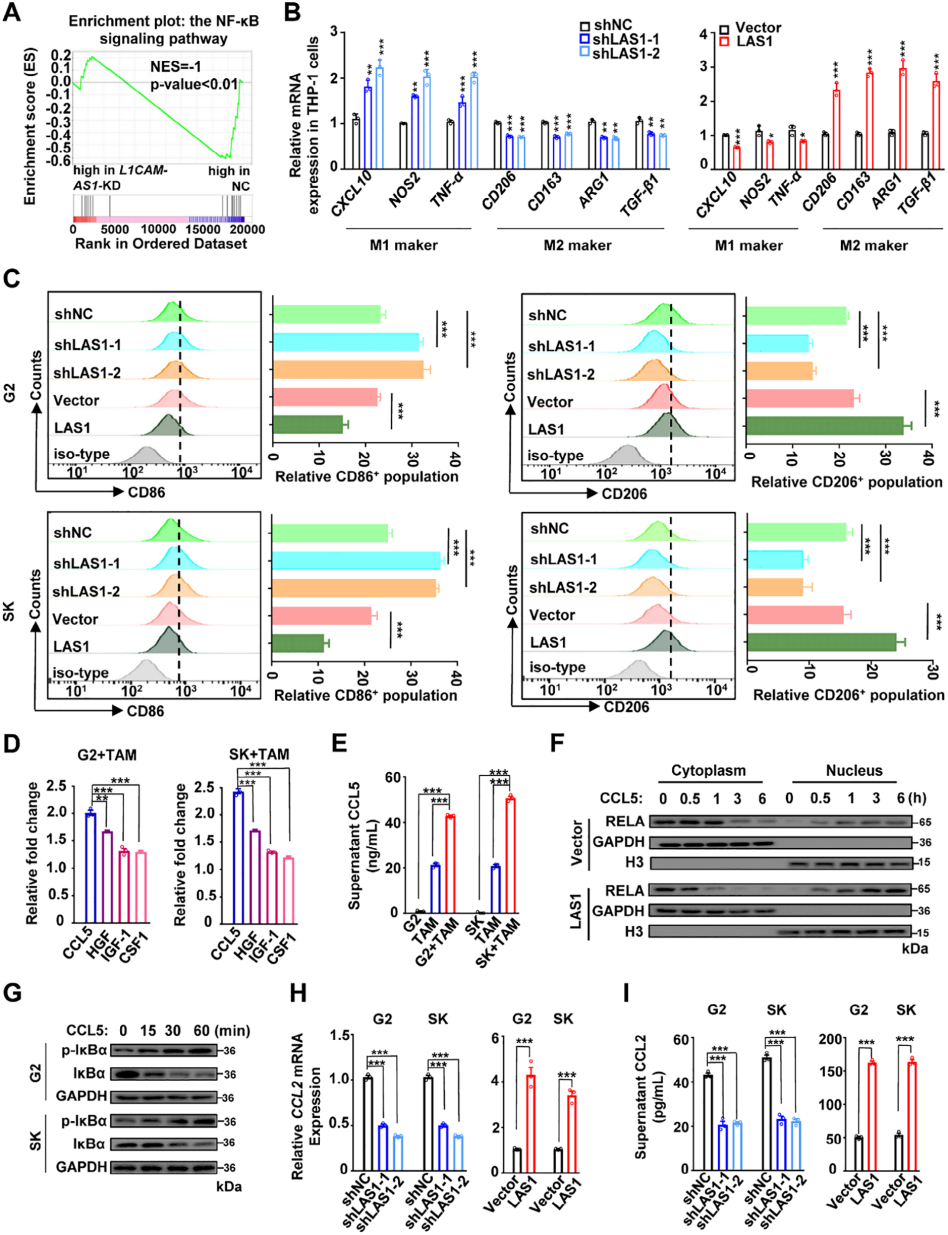
L1CAM‐AS1 facilitates RAN‐mediated RELA nuclear import, enhances CCL2 secretion of HCC cells and promotes M2 polarization of macrophages. A) GSEA analyses for dysregulated genes in *L1CAM‐AS1*‐KD G2 cells showed significant enrichments of the NF‐κB signaling pathway. B) Levels of M1 markers (*CXCL10*, *NOS2*, and *TNF‐α*) and M2 markers (*CD206*, *CD163*, *ARG1*, and *TGF‐β1*) in THP‐1 cells co‐cultured with the *L1CAM‐AS1*‐KD or *L1CAM‐AS1*‐OE G2 cells. C) Flow cytometry (FCM) analyses of the CD86 and CD206 levels on THP‐1 cells co‐cultured with the *L1CAM‐AS1*‐KD or *L1CAM‐AS1*‐OE G2 cells. D) Among CCL5, CSF1, IGF1, and HGF, CCL5 showed most significant upregulation in the coculture medium of THP‐1 cells co‐cultured with HCC cells compared to the monoculture medium of THP‐1 cells. E) ELISA assays indicated that there were higher levels of CCL5 in the coculture medium of THP‐1 cells with HCC cells than those in monoculture medium of THP‐1 or HCC cells. F) Treatment of G2 cells with recombinant protein CCL5 increased RELA nuclear translocation. This was more evident in the *L1CAM‐AS1*‐OE G2 cells. G) The recombinant protein CCL5 elevated the p‐IκBα levels and reduced IκBα expression in HCC cells. H,I) L1CAM‐AS1 enhanced *CCL2* expression levels in HCC cells and tumor‐derived CCL2 release. Data information: Each value represents mean ± SD. The difference between two groups was calculated using Student's *t* test. *p *<* 0.05; **p *<* 0.01; ***p *<* 0.001. Data show one representative of three independent experiments with three biological replicates.

GSEA of the *L1CAM‐AS1*‐KD HepG2 cell RNA‐seq profiling data showed significant enrichments of the NF‐κB signaling pathway (**Figure** **5**A). Considering that the crucial involvement of the NF‐κB pathway in anti‐tumor immunity, we hypothesized that the L1CAM‐AS1‐RAN axis might contribute to HCC development through regulating TME. To test it, we firstly assessed correlations between the *RAN* levels and infiltration levels of 22 types of immune cells using the TCGA LIHC data via CIBERSORT. The immunogenomic analyses revealed a significant association of *RAN* expression with TAM infiltrations (Figure S6A, Supporting Information). We then successfully validated the impacts of the L1CAM‐AS1‐RAN axis in HCC cells on M2‐like polarization of TAMs (Figure 5B,C and Figure S6B, Supporting Information). There were evidently increased levels of M1 markers (such as *CXCL10*, *NOS2*, and *TNF‐α*) and reduced levels of M2 markers (such as *CD206*, *CD163*, *ARG1*, and *TGF‐β1*) in the THP‐1 cells co‐cultured with the *L1CAM‐AS1*‐KD HCC cells (Figure 5B and Figure S6B, Supporting Information). On the contrary, the THP‐1 cells co‐cultured with the *L1CAM‐AS1*‐OE cells showed downregulated M1 markers and evaluated M2 markers (Figure 5B and Figure S6B, Supporting Information). Subsequent FCM analyses verified the impacts of L1CAM‐AS1 in neoplastic cells on macrophage M2‐polarization (CD86 low/CD206 high) (Figure 5C). Similarly, *RAN* in HCC cells markedly enhanced M2 polarization and inhibited M1 polarization of macrophages (Figure S7A–D, Supporting Information). Together, these results demonstrated that the L1CAM‐AS1‐RAN axis regulates TME via enhancing M2 polarization of TAMs.

### 2.7 L1CAM‐AS1 Facilitated RAN‐Mediated RELA Nuclear Import in HCC Cells and CCL2 Secretion

Considering that RELA (p65) is transported into the nucleus in a RAN‐dependent manner,^[^ ^14^ ^]^ we hypothesized that L1CAM‐AS1 may modulate RELA nuclear import by regulating RAN expression. Our previous data indicated that L1CAM‐AS1 promotes M2 polarization, whereas the M2 macrophages in TME can release CCL5, CSF1, IGF1 and HGF to increases the immunosuppressive environment and promote tumor cell proliferation.^[^ ^11^, ^15^, ^16^ ^]^ As a result, we detected levels of CCL5, CSF1, IGF1 and HGF in the culture medium of THP‐1 cells with or without HCC cells. Among them, levels of CCL5 were most significantly upregulated in the coculture medium compared to the monoculture medium of macrophages (Figure 5D and Figure S6C, Supporting Information). Meanwhile, much higher CCL5 levels were observed in the coculture medium of macrophages and HCC cells than in monoculture medium of macrophages or HCC cells (Figure 5E), suggesting that CCL5 in TME is mainly from macrophages. Treatment with recombinant protein CCL5 increased RELA nuclear translocation in both HepG2 and SK‐HEP‐1 cells, which was more evident in the *L1CAM‐AS1*‐OE cells (Figure 5F and Figure S6D, Supporting Information). However, when the *L1CAM‐AS1*‐KD cells were treated with CCL5, the nuclear RELA levels were reduced in HCC cells (Figure S6E, Supporting Information), indicating that L1CAM‐AS1 enhances CCL5‐induced RELA nuclear translocation.

It has been found that CCL5 treatment induces elevated levels of IκBα phosphorylation (p‐IκBα), ubiquitin‐related degradation of IκBα, elevated phosphorylation of RELA in the cytosol and, thus, translocation of RELA into the nucleus in lung cancer or colorectal cancer cells.^[^ ^17^, ^18^ ^]^ Consistently, the recombinant protein CCL5 increased the p‐IκBα levels as well as decreased IκBα expression in HCC cells (Figure 5G). We further examined whether CCL5 regulates nuclear translocation of RELA is RAN‐dependent in HCC cells. After knocking down RAN in HCC cells, we observed inhibition of RELA nuclear import (Figure S8A,B, Supporting Information). However, ectopic *RAN* expression accelerated translocation of RELA to nucleus (Figure S8C, Supporting Information). Furthermore, we performed rescue assays to validate the role of the *L1CAM‐AS1*‐RAN axis in regulating CCL5‐induced nuclear translocation of RELA in cells (Figure S8D, Supporting Information). Our results indicated that knockdown of *RAN* inhibited the increased nuclear translocation of RELA due to overexpression of L1CAM‐AS1 in HCC cells (Figure S8D, Supporting Information). Next, we explored the underlying mechanism of L1CAM‐AS1‐induced activation of NF‐κB signaling in HCC cells. Emerging evidence indicated that CCL2 is overexpressed in HCC and promotes M2‐polarisation of TAMs, resulting in the immunosuppression status of TME.^[^ ^12^ ^]^ Indeed, *CCL2* in the NF‐κB signaling was identified according to GSEA analyses and its expression was suppressed (Figure 5A and Figure S8E, Supporting Information). Consistently, knockdown of *L1CAM‐AS1* or *RAN* reduced expression levels of *CCL2* in HCC cells (Figure 5H and Figure S8E, Supporting Information); whereas, ectopic *L1CAM‐AS1* or *RAN* expression enhanced *CCL2* expression levels (Figure 5H and Figure S8F, Supporting Information). We further examined tumor‐derived CCL2 release in culture medium of HCC cells using ELISA and found consistent results to that of *CCL2* mRNA levels (Figure 5I and Figure S8G, Supporting Information). These findings indicated that L1CAM‐AS1 promoted HCC‐derived CCL2 secretion via upregulation of the RAN‐mediated RELA nuclear import.

### 2.8 Loss of Ran Induced Anti‐Tumor Immunity and Sensitized Immunotherapy against Liver Cancer in Mice

**Figure 6:**
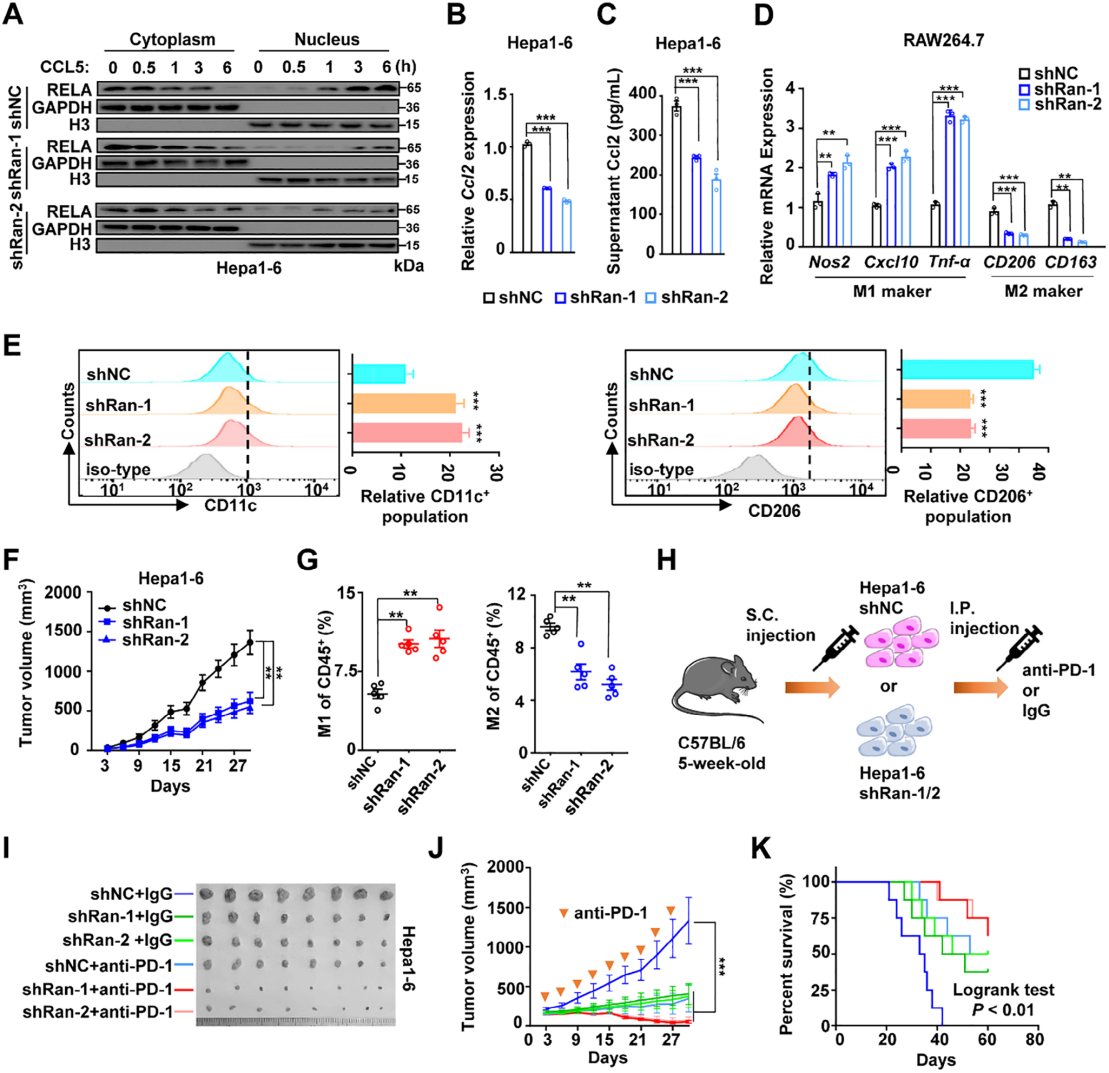
Loss of *Ran* enhances anti‐tumor immunity against HCC in mice. A) Silencing of *Ran* reduced nuclear import of RELA in Hepa1‐6 cells treated with Ccl5. B,C) Silencing of *Ran* suppressed *Ccl2* expression in Hepa1‐6 cells and tumor‐derived Ccl2 secretion. D) Levels of M1 markers (*Nos2*, *Cxcl10*, and *Tnf‐α*) and M2 markers (*Cd206* and *Cd163*) in RAW264.7 cells co‐cultured with the *Ran*‐KD Hepa1‐6 cells. (E) FCM analyses of the Cd11C and Cd206 levels on RAW264.7 cells co‐cultured with the *Ran*‐KD Hepa1‐6 cells. F) Silencing of *Ran* inhibited proliferation of the Hepa1‐6 xenografts. G) Compared to the control xenografts, there were elevated levels of M1 macrophages but decreased amounts of M2 macrophages in the *Ran*‐KD tumors in mice. H) Schematic diagram of the anti‐PD‐1 treatment schedule for mice bearing *Ran*‐KD xenografts. I) The subcutaneous HCC xenografts from the indicated treatment groups. J) Tumor growth of mice with the tumors with or without silencing of *Ran* treated with IgG or the anti‐PD1 antibody. K) Kaplan–Meier curve of mice with the tumors with or without silencing of *Ran* treated with IgG or anti‐PD1 antibody. Data information: Each value represents mean ± SD. The difference between two groups was calculated using Student's *t* test. One‐way ANOVA analysis with Dunnett's test was used for multiple comparisons. *p *<* 0.05; **p *<* 0.01; ***p *<* 0.001. Data show one representative of three independent experiments with three biological replicates.

To investigate the role of the L1CAM‐AS1‐RAN axis‐mediated anti‐tumor immunity in vivo, we developed multiple mouse Hepa1‐6 cell models with stably silenced *Ran* (the *Ran*‐KD cells, shRan‐1 and shRan‐2) since there is no homologous gene of human *L1CAM‐AS1* in mice (Figure S9A, Supporting Information). Loss of *Ran* reduced nuclear import of RELA in Hepa1‐6 cells treated with Ccl5, which is in line with the results human cells (**Figure** **6**A). As a result, we found that silencing *Ran* suppressed *Ccl2* expression in HCC cells as well as tumor‐derived Ccl2 secretion (Figure 6B‐C). To examine whether Ran is also involved in M2‐like polarization of mouse macrophages, we measured M1 and M2 markers of RAW264.7 cells co‐cultured with the *Ran*‐KD Hepa1‐6 cells and observed increased M1 markers (*Nos2*, *Cxcl10*, and *Tnf‐α*) and decreased M2 markers (*Cd206* and *Cd163*) in macrophages (Figure 6D). Consistently, the FCM analyses demonstrated that the percentage of CD11c positive cells was significantly increased in macrophages cocultured with the *Ran*‐KD Hepa1‐6 cells, whereas the percentage of CD206 positive cells was decreased in macrophages (Figure 6E). Meanwhile, RAW264.7 macrophages co‐cultured with Hepa1‐6 cells showed increased CCL5 secretion (Figure S9B, Supporting Information). We further validated these findings in vivo through establishing the Hepa1‐6 xenograft mouse model in C57BL/6 mice. We found that proliferation of the *Ran*‐KD xenografts was significantly suppressed compared to controls (Figure 6F and Figure S9C,D, Supporting Information). Intriguingly, we found increased amounts of M1 TAMs but decreased amounts of total TAMs and M2 TAMs in the *Ran*‐KD xenografts compared to the control xenografts (Figure 6G and Figure S9E,F, Supporting Information). To further explore the role of Ran in enhancing macrophage M2 polarization, we deplete macrophages in mice using anti‐CSF1R antibodies prior to tumor inoculation and found that depletion of macrophages rescued the anti‐tumor effects of the *Ran*‐KD xenografts (Figure S9H,I, Supporting Information). Collectively, these data indicated that Ran also play a pivotal role in reprogramming HCC TME via enhancing M2‐polarization of macrophages.

**Figure 7:**
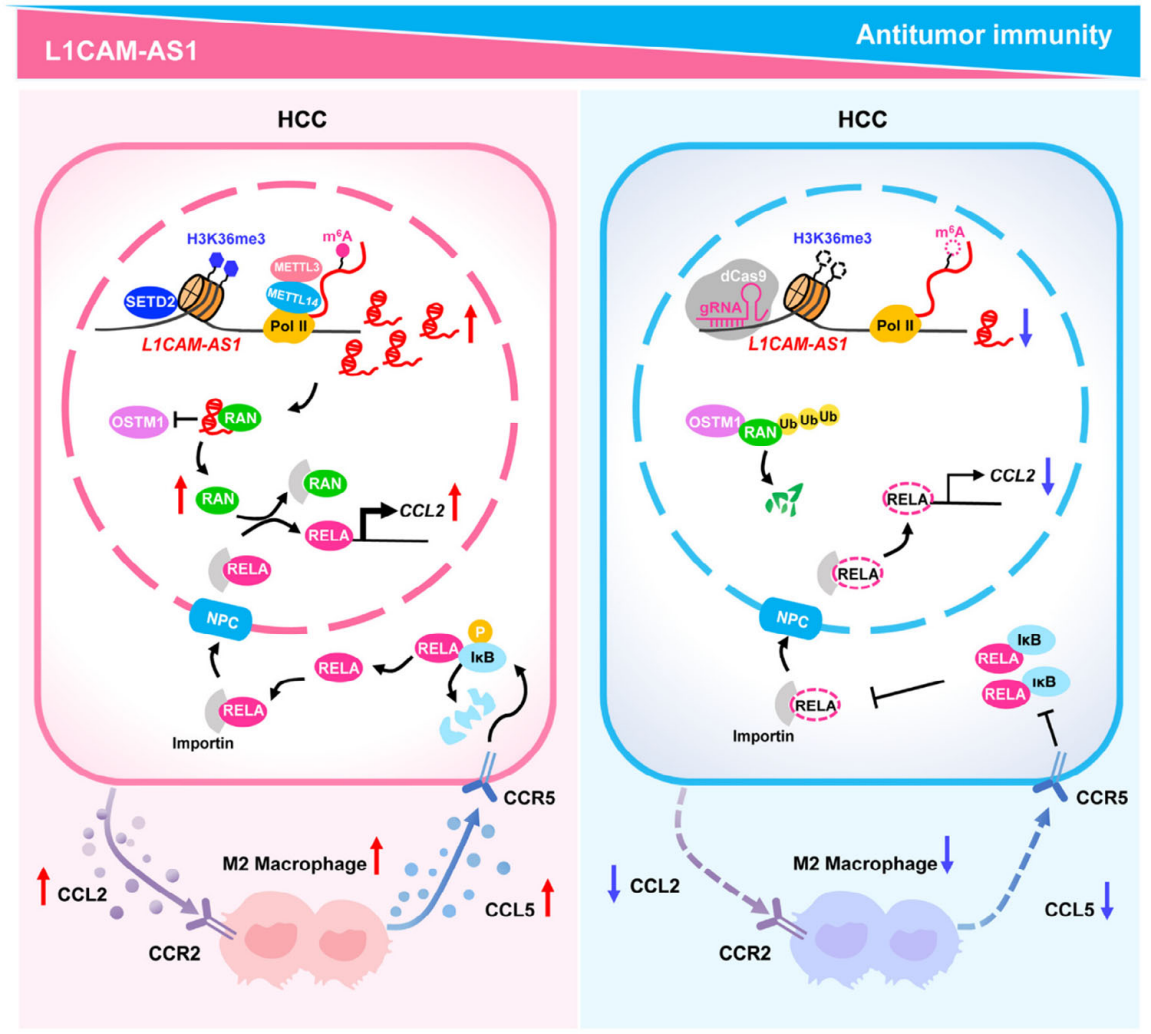
Graphical representation of the regulation and functions of *L1CAM‐AS1*‐RAN axis in antitumor immunity of HCC. Oncogenic L1CAM‐AS1 is a novel H3K36me3‐guided, m^6^A‐modified lncRNA in HCC. L1CAM‐AS1 stabilizes RAN protein, activates the NF‐κB signaling and up‐regulates expression of *CCL2* via promoting nuclear import of transcription factor RELA (p65). *L1CAM‐AS1*‐induced CCL2 release by HCC cells promoted M2 polarization of macrophages, which suppressed antitumor responses. Meanwhile, immunosuppressive M2 TAMs‐secreted CCL5 enhanced RELA nuclear import and activated the NF‐κB proinflammatory pathway in HCC cells. Inhibition of the *L1CAM‐AS1*‐RAN axis promoted the anticancer efficacy of PD‐1 blockade via TAM reprogramming in mouse HCC models.

Only small portion of HCC patients have response to PD‐1‐based immunotherapy.^[^ ^11^ ^]^ As showed previously, high levels of Ran in HCC cells enhanced the immunosuppressive TME and suppressed antitumor immunity, which led to a hypothesis that Ran inhibition is a probable way to sensitize anti‐PD‐1 therapy. To test this, we gave anti‐PD‐1 treatment (200µg per mouse) to mice bearing Hepa1‐6 tumors with or without *Ran* depletion (Figure 6H). Although either loss of *Ran* or anti‐PD‐1 treatment significantly reduced tumorigenicity as compared with the controls in Hepa1‐6 tumor bearing mice, these single treatments alone were unable to eradicate the tumors (Figure 6I,J and Figure S9G–I, Supporting Information). Notably, mice in the *Ran* depletion group treated with the anti‐PD‐1 antibody showed an evident tumor control, a minimized tumor volume, and an evidently prolonged survival time (Figure 6I–K and Figure S9G–I, Supporting Information). To verify results of the subcutaneous tumor model, we established an orthotopic HCC model in mice and similar results were observed (Figure S9J, Supporting Information). Altogether, these data demonstrated that targeted Ran inhibition enhances the efficacy of anti‐PD‐1 in HCC via abrogation of M2‐like TAMs‐included immunosuppression (**Figure** **7**).

## 3 Discussion

It remains poorly understood how H3K36me3‐guided m^6^A methylation regulates lncRNA expression. In the current study, we bridge this gap by identifying *L1CAM‐AS1* as a novel H3K36me3‐guided, m^6^A‐modified lncRNA in HCC. Oncogenic L1CAM‐AS1 stabilizes RAN protein, upregulates RAN expression, promotes RAN‐mediated nuclear import of RELA (p65), increases RELA‐controlled *CCL2* expression and activates the NF‐κB signaling in HCC cells. This leads to elevated CCL2 secretion from HCC cells and M2 polarization of TAMs, whereas M2 TAMs‐released CCL5 augments RELA nuclear import in HCC cells. Inhibited L1CAM‐AS1‐RAN axis leads to obviously improved efficacies of PD‐1 blockade in HCC mouse models.

Histone modification H3K36me3 marks are involved in regulating transcriptional activity, transcription elongation, pre‐mRNA processing, and alternative splicing.^[^ ^3^, ^19^, ^20^ ^]^ Besides protein‐coding genes, the density of H3K36me3 also correlates well with expression of lncRNA genes in different cell types and tissue types,^[^ ^21^ ^]^ elucidating its role in regulating lncRNA expression. Previous investigations focused on how H3K36me3 impacts lncRNA transcription. For instance, depletion of the Pol‐II‐associated transcription elongation factor SPT6 promoted One example is a rearrangement of H3K36me3 histone marks from active protein‐coding to lncRNA genes, which led to enhanced lncRNA transcription, when the Pol‐II‐associated transcription elongation factor SPT6 is depleted.^[^ ^22^ ^]^ Additionally, JMJD2C protein regulated the H3K36me3 levels of *MALAT1* promoter and modulated lncRNA MALAT1 expression in CRC.^[^ ^23^ ^]^ By contrast, we found that H3K36me3 marks could induce lncRNA m^6^A modification and, thus, elevated expression, with *L1CAM‐AS1* as an example in HCC. As a result, our data provide novel insights into complicated relationships of various epigenetic alterations (H3K36me3, m^6^A modification, and lncRNAs) in cells.

As a highly conserved GTPase predominantly localized in the nucleus when bound to GTP, RAN is overexpressed in malignancies.^[^ ^24^, ^25^ ^]^ The nuclear import processes mediated by importins are fine‐regulated by the small GTPase RAN.^[^ ^24^, ^25^ ^]^ Indeed, oncogenic Ran is involved in stearoyl‐CoA desaturase‐induced HCC development in mice through reducing nuclear import of HuR and increasing HuR‐mediated stabilization of *LRP5* and *LRP6* mRNAs.^[^ ^26^ ^]^ Additionally, the molecular 5‐gene score (*RAN*, *HN1*, *RAMP3*, *KRT19*, and *TAF9*) is associated with outcomes of HCC patients after resection, indicating the clinical potential of RAN as HCC prognostic markers.^[^ ^27^ ^]^ However, the underlying mechanisms of RAN ubiquitination in cells have remained poorly defined. We first report that L1CAM‐AS1 can inhibit RAN ubiquitination, reduce RAN proteasome‐degradation, and upregulate RAN expression in HCC. Our data develop the knowledge about the implications of ncRNA‐regulated ubiquitination of the small GTPase RAN during HCC development.

When nuclear localization signal (NLS)‐containing proteins are imported into the nucleus, importins firstly recognize these substrates in the cytoplasm and facilitate their transport through the nuclear pore complex (NPC) into the nucleus. Next, RAN binds to the substrate‐importin complexes in the nucleus, which triggering the release of imported cargoes.^[^ ^24^, ^25^ ^]^ Interestingly, degradation of IκBα unmasks the NLS of RELA (p65) in the cytosol, which leads to binding of importins with RELA and nuclear import of RELA.^[^ ^14^ ^]^ The presence of the small GTPase RAN in the nucleus released RELA from importins and activation of the NF‐κB signaling.^[^ ^14^ ^]^ Consistently, we for the first time demonstrated that the L1CAM‐AS1‐RAN axis could enhance RELA nuclear import, boost *CCL2* transcription and elevate CCL2 secretion in HCC cells, which establishes a potent paracrine regulatory circuit for macrophage M2 polarization and immunosuppression. Our findings extend the understanding of mechanisms underlying dysregulated lncRNAs in anti‐tumor immune responses.

CCL5 could contribute to malignant proliferation, invasion, metastasis, and immunosuppressive TME formation.^[^ ^16^, ^28^, ^29^ ^]^ Emerging evidence suggests that TAMs are a major source of CCL5, which promotes tumor progression via CC receptor 5 (CCR5) in pancreatic cancer, gastric cancer and glioma.^[^ ^16^, ^30^, ^31^ ^]^ However, the role of CCL5 from macrophages in HCC progression remains elusive. In this study, we found that HCC cells upregulated CCL5 release from macrophages via a CCL2‐mediated paracrine regulatory circuit. Macrophage‐derived CCL5 promoted HCC development by elevating the levels p‐IκBα, reducing IκBα expression and activating nuclear import of RELA in HCC cells. Together, our findings support that HCC cells activate M2 polarization of TAMs via CCL2 and macrophages promote cancer progression via CCL5.

In summary, we identified H3K36me3‐guided, m^6^A‐modified *L1CAM‐AS1* as a novel oncogene in HCC. On one hand, L1CAM‐AS1 acts through stabilizing and elevating onco‐protein RAN, leading to enhanced proliferation and metastasis of HCC cells. On the other hand, L1CAM‐AS1 facilitates the nuclear import of RELA (p65) by increasing RAN expression, enhances CCL2 release of cancer cells, induces M2‐like macrophages, thereby forming immunosuppressive TME. These insights highlight a hitherto unrecognized mechanism of epigenetic alternations in HCC driving the crosstalk between malignant cells and macrophages via the CCL2/CCL5 positive feedback regulation. Our findings also imply the clinical potentials of the L1CAM‐AS1‐RAN axis as novel biomarkers and the importance of targeting this signaling to increase sensitivity to ICIs among HCC patients.

## 4 Experimental Section

### Cell Culture

Human HepG2, SK‐HEP‐1, and HEK293T cells as well as mouse Hepa1‐6 cells were cultured in DMEM medium (Gibco, USA). Human THP‐1 Cells were cultured in RPMI 1640 medium (Gibco, USA). HUVEC cells were cultured in ECM medium (ScienCell, USA). All media were supplemented with 10% fetal bovine serum (FBS; Gibco, 1347575). HepG2, SK‐HEP‐1, THP‐1 and Hepa1‐6 cells were obtained from the Cell Bank of Type Culture, Chinese Academy of Sciences (Shanghai). HEK293T cells were kindly provided by Dr. Yunshan Wang (Jinan Central Hospital, Shandong Province, China). HUVEC cells were obtained from ScienCell company. Cells were maintained at 37 °C in a 5% CO_2_ incubator and routinely tested to verify that they were mycoplasma‐negative as reported previously.^[^ ^32^, ^33^, ^34^, ^35^, ^36^ ^]^

### Chromatin Immunoprecipitation Sequencing (ChIP‐Seq) and ChIP‐Quantitative PCR (ChIP‐qPCR)

The ChIP assays were performed using a total of 2.5 × 10^7^ HepG2 or SK‐HEP‐1 cells with 5 µg of the relevant antibodies as previously described.^[^ ^33^ ^]^ For the ChIP‐seq assay, the Illumina NovaSeq 6000 platform was utilized to sequence the immunoprecipitated DNA. For ChIP‐qPCR assays, the fold enrichment of purified ChIP DNA relative to input DNA at a given genomic site was determined with ChIP‐qPCR primers (Table S1, Supporting Information).

### m^6^A RNA Immunoprecipitation (MeRIP) and RIP‐qPCR

As previously reported, all MeRIP and RIP assays were conducted using the designated antibodies or the IgG isotype‐control (Table S2, Supporting Information).^[^ ^32^, ^33^, ^35^ ^]^ The lncRNA levels in the precipitates were measure using RT‐qPCR.

### Quantitative Reverse Transcription PCR (RT‐qPCR)

Reverse transcribed total RNA was used to determine the relative expression of candidate genes with the indicated primers (Table S1, Supporting Information) as previously reported.^[^ ^32^, ^33^, ^34^, ^35^, ^36^ ^]^ Melt curve analyses were used to verify the specificity of the PCR products.

### Cell Transfection

The negative control RNA duplex (NC) and small interfering RNA (siRNA) duplexes for *SETD2*, *METTL3*, *METTL14*, *IGF2BP1*, *IGF2BP2*, *IGF2BP3*, *RAN*, *OSTM1*, *RELA*, and *Ran* genes were products of Genepharma (Shanghai, China) (Table S3, Supporting Information). All siRNAs were transfected with INTERFERin (Polyplus, 409‐10) and all plasmids were transfected by with jetPRIME (Polyplus, 114‐07).

### CRISPR/dCas9‐KRAB Interference (dCas9‐CRISPRi) and CRISPR/dCas13b‐ALKBH5 Interference (dCas13b‐CRISPRi)

For dCas9‐CRISPRi assays, four guide RNAs (gRNAs) targeting the *L1CAM‐AS1* H3K36me3 modification sites were designed (Table S3, Supporting Information). The gRNA DNA templates were cloned into the pLV hU6‐sgRNA hUbC‐dCas9‐KRAB‐T2a‐GFP vector (Addgene, #71237) as previously described.^[^ ^33^ ^]^ Following transfection of the recombinant plasmids into HCC cells, *L1CAM‐AS1* expression was detected through RT‐qPCR. For dCas13b‐CRISPRi assays, two gRNAs adjacent to the L1CAM‐AS1 m^6^A site were designed (Table S3, Supporting Information). The gRNA DNA templates were cloned into the pLKO.1 vector. After the recombinant constructs were co‐transfected with pcDNA‐dCas13b‐ALKBH5 into cells, m^6^A or expression levels of L1CAM‐AS1 were measured through MeRIP‐qPCR or RT‐qPCR as previously described.^[^ ^37^ ^]^

### Patients and Tissue Specimens

One hundred and ninety HCC patients (Shandong cohort, *n* = 77; Jiangsu cohort, *n* = 113) were recruited between April 2009 and July 2019 in this study. The detailed characteristics of most HCC cases were reported previously.^[^ ^33^ ^]^

### The Plasmid Constructs

The human *L1CAM‐AS1* cDNA (NR_130768.1), containing a tag sequence (5′‐GTCGTATCCAGTGCGAATACCTCGGACCCTGCACTGGATACGAC‐3′) at its 3′‐end, was synthesized and cloned into pcDNA3.1 by Genewiz (Suzhou, China). The plasmid was named as m^6^A‐WT. The three mutants (Mut 1, Mut 2, and Mut 3) of m^6^A‐WT were constructs with the A‐to‐G mutation at the 286, 430, or 436 base of the *L1CAM‐AS1* cDNA. Human *L1CAM‐AS1* cDNA was cloned into the pCDH‐CMV‐MCS‐EF1α‐Puro vector, and the resultant plasmid was designated LAS1. Two *L1CAM‐AS1* shRNAs (shLAS1‐1 or shLAS1‐2) and a negative control shRNA (shNC) were synthesized and cloned into pLKO.1 (Table S3, Supporting Information). The resulting plasmids were named as shLAS1‐1, shLAS1‐2, and shNC. The full‐length *L1CAM‐AS1* cDNA with the T7 promoter inserted upstream and downstream from the cloning site was also cloned into pcDNA3.1. The plasmid was designated LAS1‐WT. Five truncated *L1CAM‐AS1* plasmids were derived from the LAS1‐WT plasmid, each with a specific deletion of 1nt‐376nt, 377nt‐593nt, 594nt‐810nt, 811nt‐1027nt, or 1028nt‐1392nt region of L1CAM‐AS1. The cDNA for the HA‐tagged *RAN* cDNA (NM_006325.5) and truncated mutants of HA‐tagged *RAN* (Δ1‐102aa and Δ103‐204aa) were cloned into pcDNA3.1. The cDNA for the Flag‐tagged *RAN* cDNA (Flag‐RAN‐WT) and three mutants (Mutant152K, Mutant167K, and Mutant152K+167K) were also cloned into pcDNA3.1. Two mice *Ran* shRNAs (shRan‐1 or shRan‐2) were synthesized and cloned into pLKO.1 (Table S3, Supporting Information). The resultant constructs were designated shRan‐1 and shRan‐2.

### Lentiviral Transduction

As previously described, recombinant lentiviral particles were produced.^[^ ^32^, ^33^, ^34^ ^]^ A viral supernatant containing 5 µg mL^−1^ polybrene was used to infect SK‐Hep‐1, HepG2, or Hepa1‐6 cells. The infected cells were then chosen using either blasticidin or puromycin at a concentration of 2 mg mL^−1^. RT‐qPCR was used to assess the *L1CAM‐AS1* or *Ran* expression in the cells.

### Cell Proliferation and Colony Formation Assays

As previously reported,^[^ ^32^ ^]^ cell proliferation and colony formation assays were performed. Stable *L1CAM‐AS1*‐knockdown (*L1CAM‐AS1*‐KD) or *L1CAM‐AS1*‐overexpression (*L1CAM‐AS1*‐OE) HepG2 or SK‐Hep‐1 cells were used in these assays.

### Wound Healing and Transwell Assays

Wound healing and transwell assays were performed s as reported previously.^[^ ^32^, ^33^, ^34^ ^]^ The impacts of *L1CAM‐AS1* on the migration and invasion capabilities of HCC cell were evaluated through these assays.

### HUVEC Tube Formation Assays

After HCC cells were cultured for 24 h, the culture medium was collected and filtered. HUVECs were seeded in a 96‐well plate coated with 50 µL of Matrigel (BD Biosciences) and cultured with the HCC cell culture medium. After 12 h, tube formation of HUVECs was observed using a microscope.

### Xenografts

To investigate the involvement of *L1CAM‐AS1* in tumor formation in vivo, the stably *L1CAM‐AS1*‐OE SK‐Hep‐1 cells were mixed with Matrigel (1:1) and injected into the right axillary fossa of 5‐week‐old male BALB/c nude mice (Vital River Laboratory, Beijing, China). Tumor volume was measured every three days.^[^ ^32^ ^]^ In order to examine the role of *L1CAM‐AS1* in hematogenous or abdominal metastases, a total of 1 × 10^7^ *L1CAM‐AS1*‐KD or *L1CAM‐AS1*‐OE SK‐Hep‐1 cells with stable firefly luciferase expression were injected into tail vein or the middle of the lower abdomen of male nude BALB/c mice (Vital River Laboratory) (*n* = 4 per group) as reported previously.^[^ ^33^, ^34^ ^]^ All mouse‐related procedures were approved by the Animal Care Committee of Shandong Cancer Hospital and Institute. All analyses were conducted in a blinded manner with individuals who were unaware of the xenograft types.

### Subcellular Fractionation

The Nuclear/Cytoplasmic Isolation Kit (Biovision, K266) was used to separately isolated the cytosolic and nuclear fractions of SK‐Hep‐1 or HepG2 cells.

### RNA Pulldown

For in vitro RNA synthesis, LAS1‐WT was used as the template as previously reported.^[^ ^32^, ^38^ ^]^ The pulldown proteins were analyzed by liquid chromatography‐tandem mass spectrometry (LS‐MS/MS) (Hoogen Biotech Co., Shanghai, China) or Western blotting.

### Western Blotting

As previously reported,^[^ ^33^, ^34^, ^39^ ^]^ Western blotting was carried out with indicated antibodies (Table S2, Supporting Information).

### Immunofluorescence and RNA FISH

Immunofluorescence and RNA FISH were performed as previously described.^[^ ^32^, ^33^, ^34^ ^]^ Cy3 channel was used to measure L1CAM‐AS1 signals. RAN or OSTM1 was detected with the indicated antibody and CoraLite488‐conjugated conjugated antibody or coraLite594‐conjugated secondary antibody (Table S2, Supporting Information). A Zeiss LSM800 confocal microscope (Zeiss, Germany) was used to view the samples.

### Immunoprecipitation‐Mass Spectrometry (IP‐MS) and Co‐IP

IP‐MS with the RAN antibody was performed to identify the potential E3 ubiquitin ligase(s) of RAN. As previously reported,^[^ ^32^, ^34^, ^39^ ^]^ Co‐IP was performed between RAN and OSTM1. The IP‐products were analyzed by Western Blotting or LS‐MS/MS (Hoogen Biotech Co. situated in Shanghai, China).

### Turnover Assays

As described previously,^[^ ^32^ ^]^ the turnover assays were conducted. The levels of RAN and GAPDH proteins were detected in HCC cells treated with cycloheximide (Chx).

### Ubiquitination Assays

Ubiquitination assays were carried out in HepG2 and SK‐HEP‐1 cells transfected with pcDNA3.1‐HA‐ubiquitin (HA‐Ub) as previously reported.^[^ ^32^, ^35^ ^]^ To isolate ubiquitinated RAN, proteins in the cell lysate were immunoprecipitated with anti‐RAN antibody and subsequently examined by Western blotting with an anti‐HA antibody.

### RNA‐Seq and Gene Set Enrichment Analysis (GSEA)

Using the Illumina NovaSeq6000 platform, RNA‐seq of the stable L1CAM‐AS1‐KD HepG2 cells (shNC, shLAS1‐1, or shLAS1‐2) was carried out. GSEA was performed for pathways annotated in the Biocarta databases using differentially expressed genes (DEGs) (log2(FC)←1.5, *p* < 0.05) in *L1CAM‐AS1*‐KD cells relative to the control cells. Gene enrichment scores were determined based on the rank of genes and gene sets.

### Cell Co‐Culture Assays

To establish a co‐culture system for macrophages and HCC cells in vitro, a 24 mm Transwell chamber with a 0.4 µm pore polycarbonate membrane (Corning, USA) was utilized. THP‐1 monocytes (1 × 10^6^ per well) were treated with 150 nmol L^−1^ PMA (MCE, USA) and induced to M0 macrophages in the upper chamber. HCC cells (5 × 10^5^ per well) were seeded into the lower chamber separately. After 24 h, the upper chamber with THP‐1 cells was then placed onto the lower chambers with HCC cells for 12 h co‐culture.

### Flow Cytometry (FCM)

To obtain a single cell suspension, cultured cells were trypsinized and resuspended in 1 × PBS, followed by filtration through a 70 µm nylon mesh. The cells were then stained with fixable viability dye (BioLegend, USA) and permeabilized using Fixation and Permeabilization Solution (BD Biosciences, USA) at 4 °C for 30 min in the dark. After washing with 1 × PBS three times, FCM was conducted on a BD FACSAria Flow Cytometer, and the data were analyzed using FlowJo software. The antibodies used during FCM are summarized in Table S2 (Supporting Information).

### Enzyme Linked Immunosorbent Assay (ELISA)

The CCL2 (MCP‐1) or CCL5 protein levels in the culture medium of human or mouse HCC cells were determined using the Human MCP‐1 ELISA Kit (Cat. KE00091; Proteintech), the Human CCL5 ELISA Kit (Cat. KE00093; Proteintech), the Mouse MCP‐1 ELISA Kit (Cat. KE10006; Proteintech), or the Mouse CCL5 ELISA Kit (Cat. KE10017; Proteintech) in accordance with the manufacturer's instructions, respectively.

### Mouse HCC Xenografts

Mouse Hepa1‐6 cells (2 × 10^6^) with or without *Ran*‐KD were inoculated into the right flank of 6‐week‐old male C57BL/6 mice. After sacrificed, the mice were immersed in 75% ethanol to ensure proper disinfection, followed by excision and enzymatic digestion of the subcutaneous tumor into a homogeneous single‐cell suspension. The resulting cell suspension was then passed through a 70 µm mesh screen to collect cells for subsequent incubation with specific antibodies. FCM was employed to measure the percentage of macrophages within TME. For the syngeneic mouse model, Hepa1‐6 cells (2 × 10^6^) with or without *Ran*‐KO were inoculated into the right flank of 6‐week‐old male C57BL/6 mice. Mice with tumors about 150–250 mm^3^ were given intraperitoneal injections of 200 mcg anti‐PD‐1 antiboty (clone RMPI‐14, BioXCell) or isotype control IgG (clone 2A3, BioXCell) three times a week. Two weeks before HCC cell injection, 500 µg anti‐CSF1R neutralizing antibody was intraperitoneally injected into the mouse every 5 days to deplete macrophages.

### Mouse Orthotopic HCC Model

For in situ HCC modeling, Hepa1‐6 cells with overexpression of luciferase (1 × 10^6^ cells per mouse) were injected into the left hepatic lobe of the 6‐week‐old C57BL/6 mice. Postoperative buprenorphine (0.1 mg kg^−1^) was administered for 72 h. Therapeutic interventions were given to mice at day 7 post‐implantation. Tumor progression was monitored via IVIS Spectrum bioluminescence imaging.

### Statistics

Student's *t* test was used to determine the difference between the two groups. One‐way ANOVA was employed for multiple comparisons. The associations between different genes were determined using Spearman's correlation analyses. Cox regression and the log‐rank test were used in survival analyses. Statistical significance was defined as a *p* value of less than 0.05. All analyses were performed with the SPSS software package (Version 16.0, SPSS Inc.) or GraphPad Prism (Version 8, GraphPad Software, Inc.).

### Data Availability

The data generated in this study are publicly available in GSA‐human database (https://ngdc.cncb.ac.cn/gsa‐human/). The data sets include the H3K36me3 ChIP‐seq (HRA007989) and the RNA‐seq (HRA007974). The data analyzed in this study were obtained from GEO at GSE243538, GSE110323, and GSE144269. The materials and the data used in the study are available upon request from the corresponding author.

### Ethics Statement

This study was approved by the Institutional Review Board of Shandong Cancer Hospital and Institute. Each participant provided written informed permission at the time of recruiting. All research was conducted in accordance with both the Declarations of Helsinki and Istanbul.

## Conflict of Interest

The authors declare no conflict of interest.

## Author Contributions

M.Y. conceived the project and supervised all experiments; N.Z. and M.Y. designed the project; T.W., L.H., Y.H., L.Z., and Y.H. performed the research; T.W., N.Z., and T.W. analyzed the data; and M.Y., T.W., and N.Z. wrote the paper. All authors read and approved the manuscript.

## Supporting information



Supporting Information

Supporting Information

## Data Availability

The data that support the findings of this study are available in the Supporting Information of this article.
