# Supplementary material for: H3K36me3‐Guided m6A Modification of Oncogenic L1CAM‐AS1 Drives Macrophage Polarization and Immunotherapy Resistance in Hepatocellular Carcinoma
Source: Adv Sci (Weinh). 2025 Jun 19;12(33):e14909. doi: 10.1002/advs.202414909 (PMC13001626; doi:10.1002/advs.202414909)
Supplement: Supplementary file 1 — Supporting Information [file ADVS-12-e14909-s001.pdf]

Supplementary Figure 1

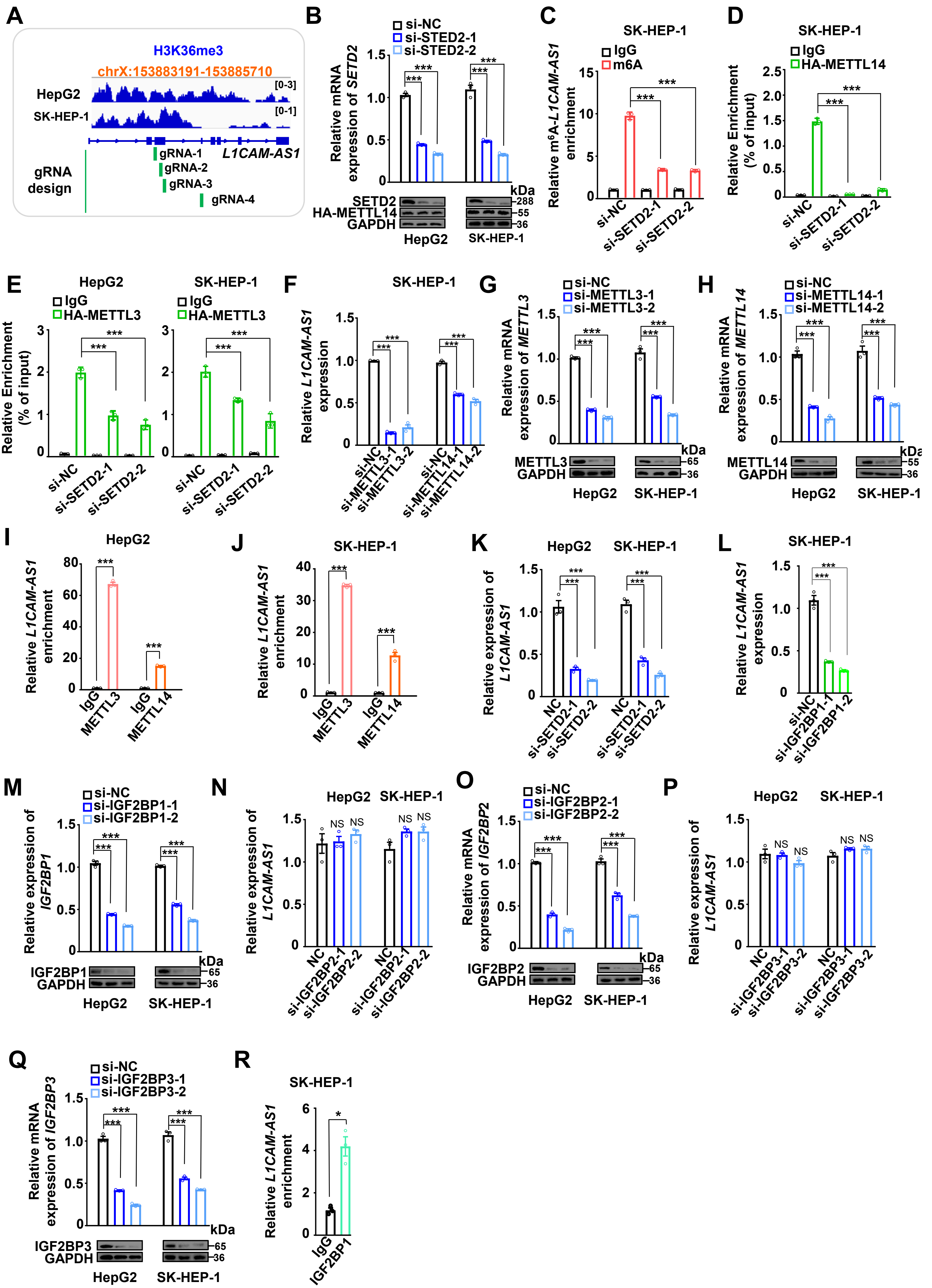

Supplementary Figure 1. *L1CAM-AS1* is a novel lncRNA with m^6A-modification guided by H3K36me3 in HCC.

(A) Schematic diagram of gRNA locations for the CRISPRi assays in HCC cells.

(B) Relative expression of *SETD2* in HepG2 and SK-HEP-1 cells with silenced *SETD2* (by siRNAs). Western blot assyas showed the SETD2 and METTL14 protein levels in cells.

(C) Knockdown of *SETD2* inhibited m^6A modification levels of L1CAM-AS1 in SK-Hep-1 cells.

(D) The ChIP-qPCR assays confirmed the decreased METTL14 enrichment levels in SK-HEP-1 cells with silenced *SETD2* (by siRNAs).

(E) ChlP-gPCR assays showed that METTL3 levels were lower in HCC cells after silencing of *SETD2*.

(F) Knock-down of *METTL3* or *METTL14* markedly downregulated L1CAM-AS1 expression in SK-HEP-1 cells.

(G) The relative expression of *METTL3* was assessed in HepG2 and SK-HEP-1 cells lines with silenced *METTL3* (by siRNAs).

(H) *METTL14* was significantly knockdown in HCC cells using siRNAs.

(I,J) The RIP-qPCR assays revealed that METTL3 and METTL14 are the writer proteins exhibiting the high binding affinities with L1CAM-AS1 in HepG2 and SK-HEP-1 cells.

(K) *SETD2* knockdown in HCC cell lines significantly suppresses L1CAM-AS1 expression.

(L) Silencing of *IGF2BP1* decreased L1CAM-AS1 expression levels in SK-HEP-1 cells.

(M) Relative expression of *IGF2BP1* in HepG2 and SK-HEP-1 cells with silenced *IGF2BP1* (by siRNAs).

(N) *IGF2BP2* was effectively silenced in HCC cells.

(O) Silencing of *IGF2BP2* did not affected L1CAM-AS1 expression levels in in HCC cells.

(P) Silencing of *IGF2BP3* had no impact on the expression levels of L1CAM-AS1 in HCC cells.

(Q) The relative expression of *IGF2BP3* was evaluated in HepG2 and SK-HEP-1 cells with silenced *IGF2BP3* (by siRNAs).

(R) The RIP assays showed association of L1CAM-AS1 with IGF2BP1 in SK-HEP-1 cells.

Data information: Each value represents mean ± SD. The difference between two groups was calculated using Student's *t* test. *$P<0.05$; **$P<0.01$; ***$P<0.001$; NS, not significant. Data show one representative of three independent experiments with three biological replicates.

Supplementary Figure 2

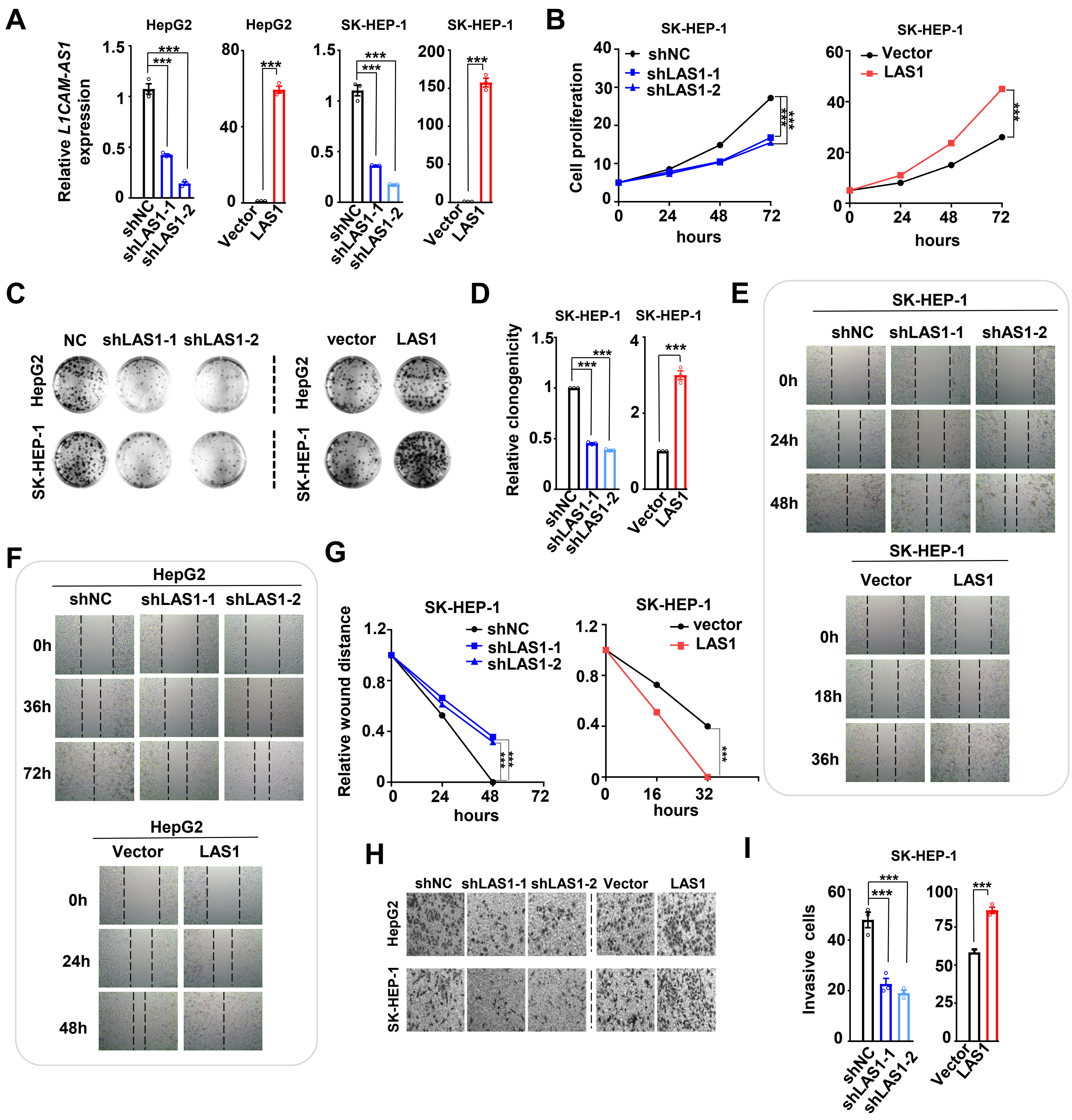

Supplementary Figure 2. *L1CAM-AS1* enhances proliferation and invasive capabilities of HCC cells.

(A) The relative expression of *L1CAM-AS1* was examined in HepG2 and SK-HEP-1 cells, where *L1CAM-AS1* was either silenced (using shRNAs) or overexpressed.

(B) Suppression of *L1CAM-AS1* in SK-HEP-1 cells significantly attenuated cell proliferation compared to the control cells, whereas stable overexpression of *L1CAM-AS1* markedly augmented proliferation of HCC cells.

(D) Clonal formation of *L1CAM-AS1*-KD and *L1CAM-AS1*-OE HCC cells.

(C) The clonogenicity of SK-HEP-1 cells was significantly attenuated by *L1CAM-AS1* knockdown, while *L1CAM-AS1* overexpression enhanced the clonogenicity of SK-HEP-1 cells.

(E, F) Representative images of wound-healing assays in *L1CAM-AS1*-KD and *L1CAM-AS1*-OE HepG2 or SK-HEP-1 cells.

(G) Knockdown of *L1CAM-AS1* in SK-HEP-1 cells impeded wound-healing, while the enforced expression of *L1CAM-AS1* significantly expedited wound-healing.

(H) The impacts of *L1CAM-AS1* knockdown or overexpression on HCC cells migration.

(I) *L1CAM-AS1* promoted migration capabilities of SK-HEP-1 cells.

Data information: The difference between two groups was calculated using Student's *t* test. One-way ANOVA analysis with Dunnett's test was used for multiple comparisons. **$P<0.01$, ***$P<0.001$, NS, not significant.

Supplementary Figure 3

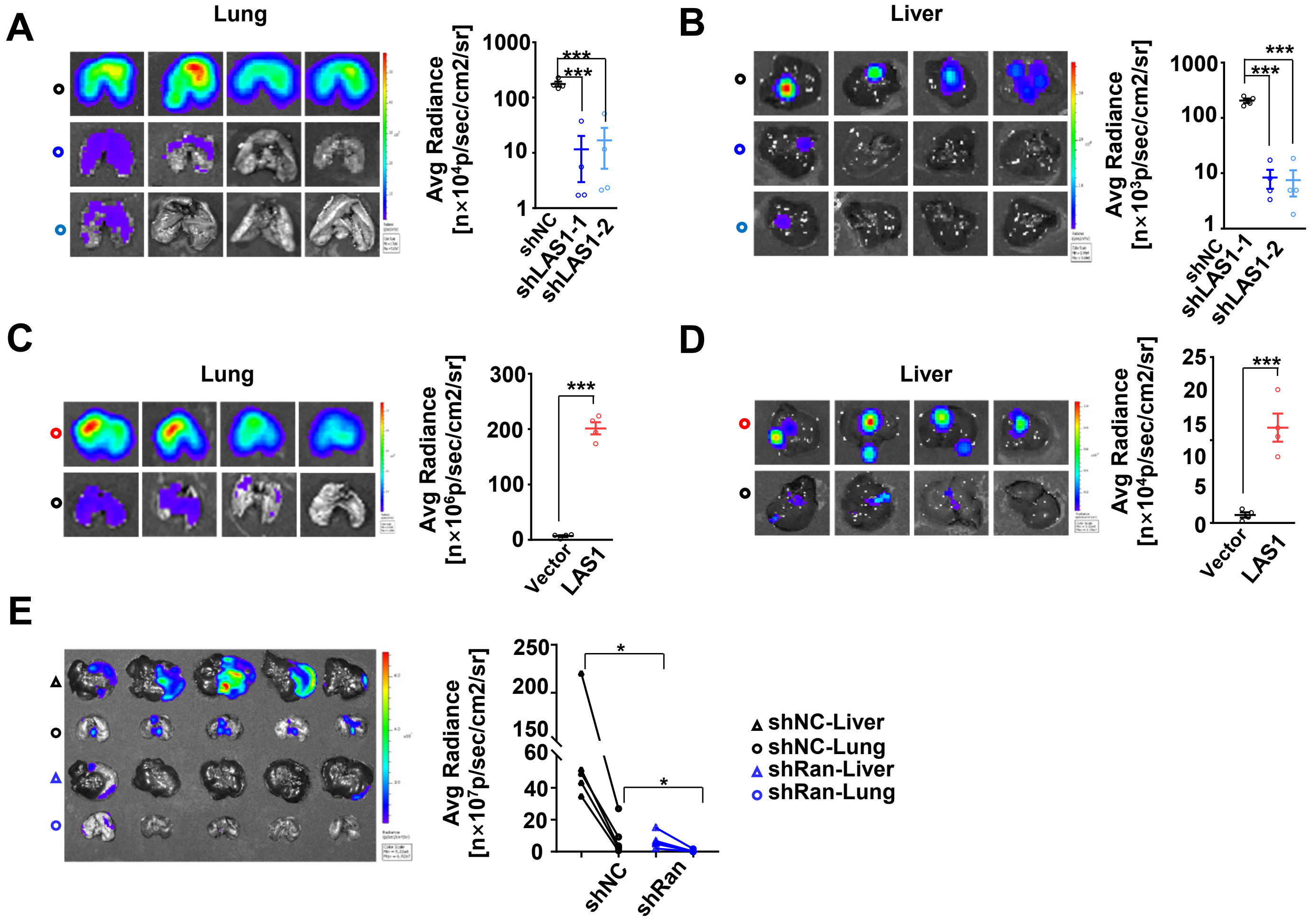

Supplementary Figure 3. *L1CAM-AS1* enhances HCC cells invasive capabilities *in vivo*.

(A) Reduced tumor metastasis was observed in the lungs of nude mice following the tail vein injection of *L1CAM-AS1*-KD SK-HEP-1 cells (n=4). The luciferase activity of cancer cells was measured on day 40 post-injection.

(B) Decreased liver tumor metastasis was observed in nude mice after the intraperitoneal injection of *L1CAM-AS1*-KD SK-HEP-1 cells (n=4). The luciferase activity of cancer cells was measured on day 35 post-injection.

(C) The injection of *L1CAM-AS1*-OE SK-HEP-1 cells resulted in an evident increase in tumor metastasis in the lungs of nude mice (n=4). Luciferase activities of cancer cells were detected on the 35th day post-injection.

(D) The overexpression of *L1CAM-AS1* resulted in an obvious increase in the liver metastasis of nude mice (n=4), with detectable luciferase activities in HCC cells 30 days post-injection.

(E) Orthotopic liver injection of the *Ran*-KD Hepa1-6 cells in C57BL/6 mice resulted in reduced primary tumor volume (n=5) and a significant decrease in pulmonary metastases

Data information: The difference between two groups was calculated using Student's *t* test. ***$P<0.001$, *$P<0.05$.

Supplementary Figure 4

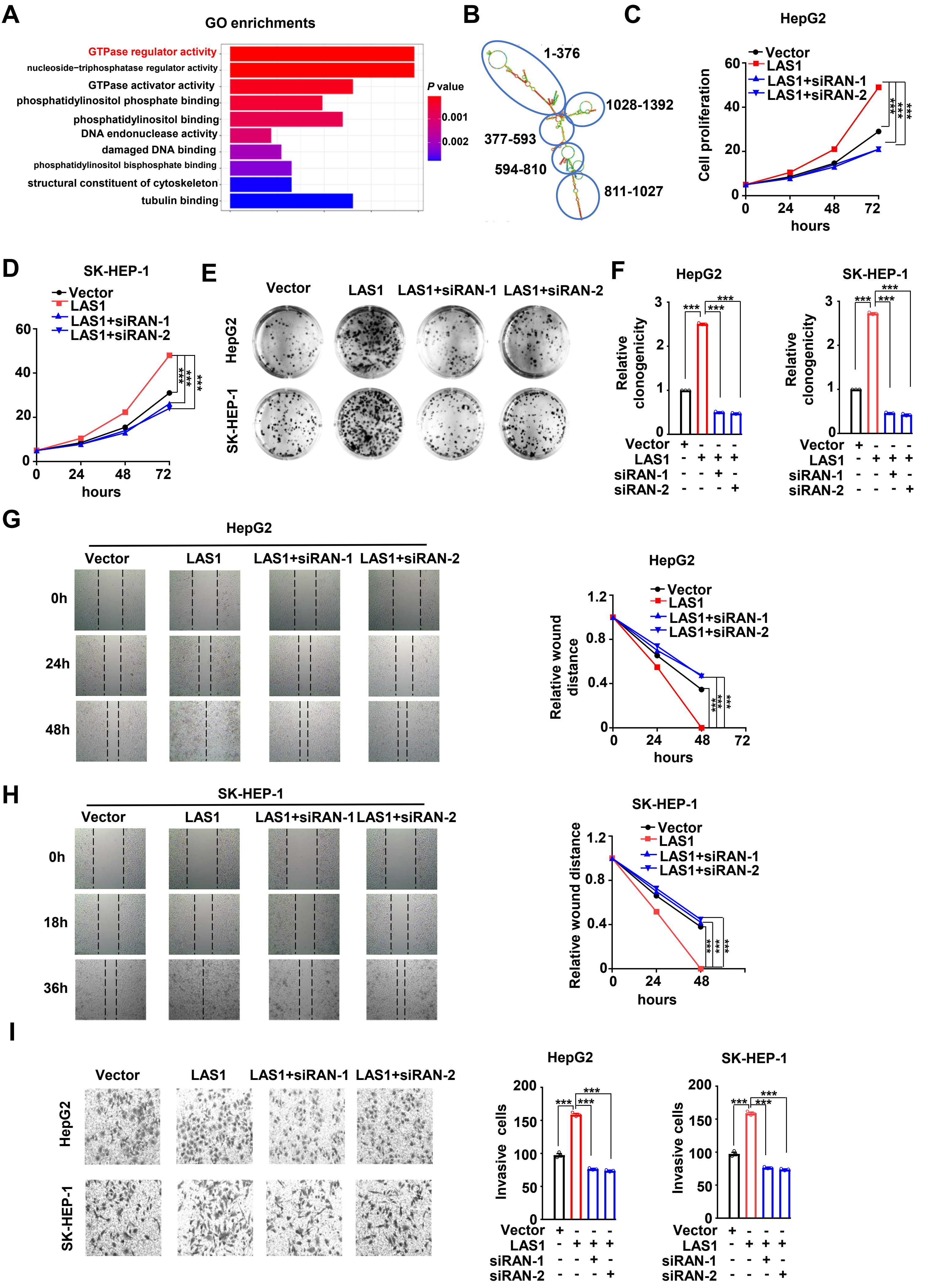

Supplementary Figure 4. The *L1CAM-AS1*-RAN axis plays a pivotal role in HCC.

(A) GO enrichment analyses of the differentially expressed genes in *L1CAM-AS1*-KD cells showed that the GTPase regulator activity pathway was significant enriched.

(B) Schematic diagram of the L1CAM-AS1 hairpin structure.

(C, D) The oncogenic functions of *L1CAM-AS1* in cell proliferation were abrogated upon knockdown of *RAN* in HCC cells.

(E, F) The oncogenic functions of *L1CAM-AS1* in clonogenicity were inhibited after silencing *RAN* in HCC cells.

(G, H) The wound-healing assays indicated that the oncogenic functions of *L1CAM-AS1* in migration were suppressed after silencing *RAN* in HCC cells.

(I) The transwell assays showed that the oncogenic functions of *L1CAM-AS1* in cell invasion were inhibited after silencing *RAN* in HCC cells.

Data information: The difference between two groups was calculated using Student's *t* test. One-way ANOVA analysis with Dunnett's test was used for multiple comparisons. *$P<0.05$, **$P<0.01$, ***$P<0.001$, NS, not significant.

Supplementary Figure 5

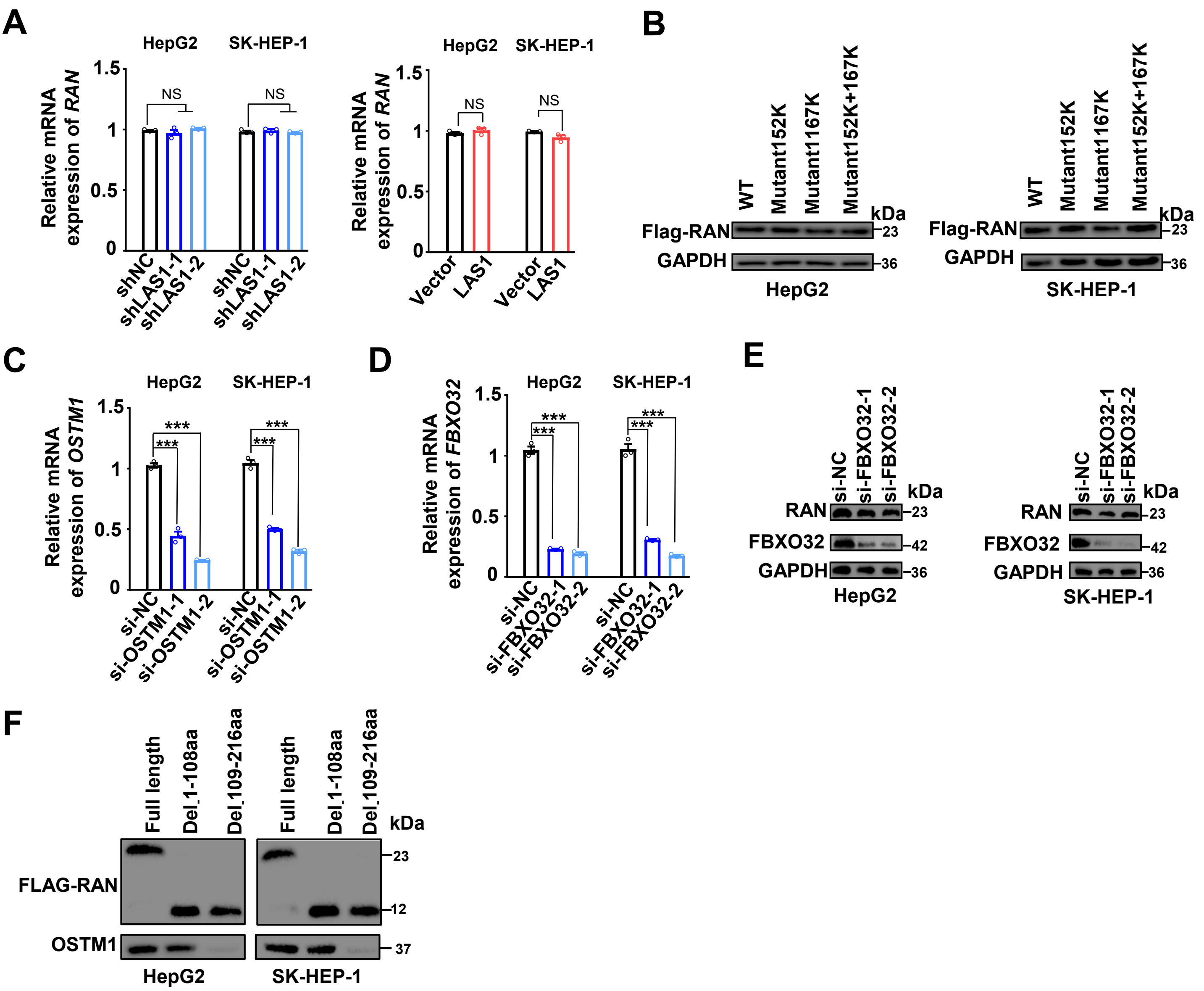

Supplementary Figure 5. OSTM1 is the E3 ubiquitin ligase of RAN protein in HCC.

(A) Knockdown of *L1CAM-AS1* or *L1CAM-AS1* overexpression did not affect mRNA levels of *RAN*.

(B) Western blot showed the levels RAN full length protein and its truncated proteins with the Flag tag in HCC cells.

(C) Relative expression of *OSTM1* in HCC cells was assessed after silencing *OSTM1* using siRNAs.

(D) Relative expression of *FBXO32* in HepG2 and SK-HEP-1 cells with silenced *FBXO32* (by siRNAs).

(E) Western blot analysis showed the protein levels of RAN and FBXO32 in HCC cells following knockdown of *FBXO32*.

(F) Co-IP assays demonstrated that OSTM1 binds to the 109-218aa region of RAN protein.

Data information: The difference between two groups was calculated using Student's *t* test. One-way ANOVA analysis with Dunnett's test was used for multiple comparisons. ****P*<0.001, NS, not significant.

Supplementary Figure 6

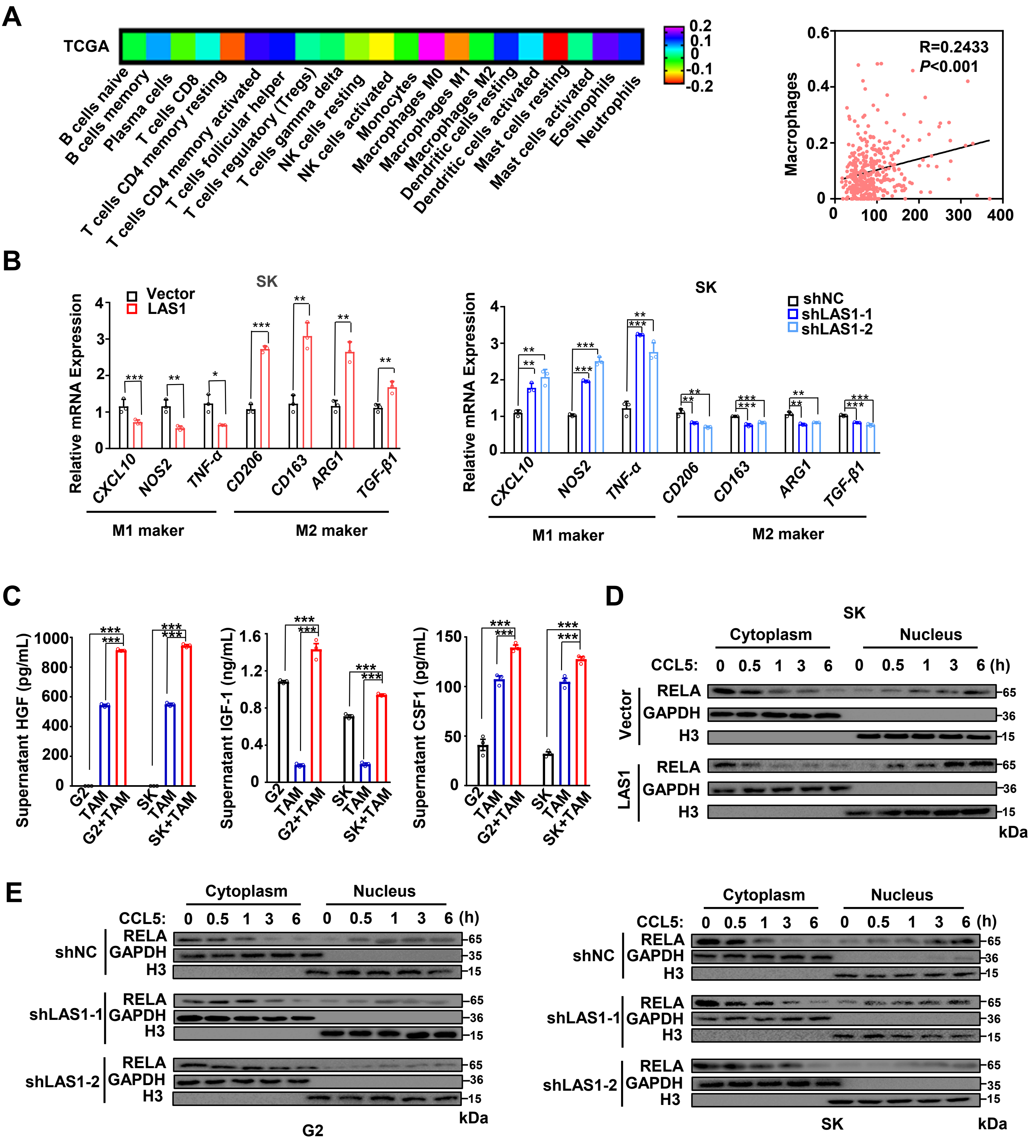

Supplementary Figure 6. *L1CAM-AS1* is implicated in enhancing M2 polarization of macrophages through modulation of RELA nuclear translocation in HCC cells.

(A) Immunogenomic analyses of the infiltration proportion of 22 immune cell types in HCC tissues with low and high *RAN* expression in TCGA-LIHC cohort using the CIBERSORT algorithm.

(B) qRT-PCR analyses of the M1 and M2 markers in THP-1 macrophages co-cultured with SK-HEP-1 cells.

(C) The levels of HGF, IGF-1 and CSF1 in the supernatants of HEPG2 or SK-HEP-1, THP-1, and co-cultures were quantified using enzyme-linked immunosorbent assays (ELISA).

(D) The overexpression of *L1CAM-AS1* promoted the nuclear entry of RELA in HCC cells induced by CCL5.

(E) The knockdown of *L1CAM-AS1* caused a significant delay in the nuclear entry of RELA in HCC cells induced by CCL5.

Data information: The difference between two groups was calculated using Student's *t* test. One-way ANOVA analysis with Dunnett's test was used for multiple comparisons. *P<0.05, **P<0.01, ***P<0.001, NS, not significant.

Supplementary Figure 7

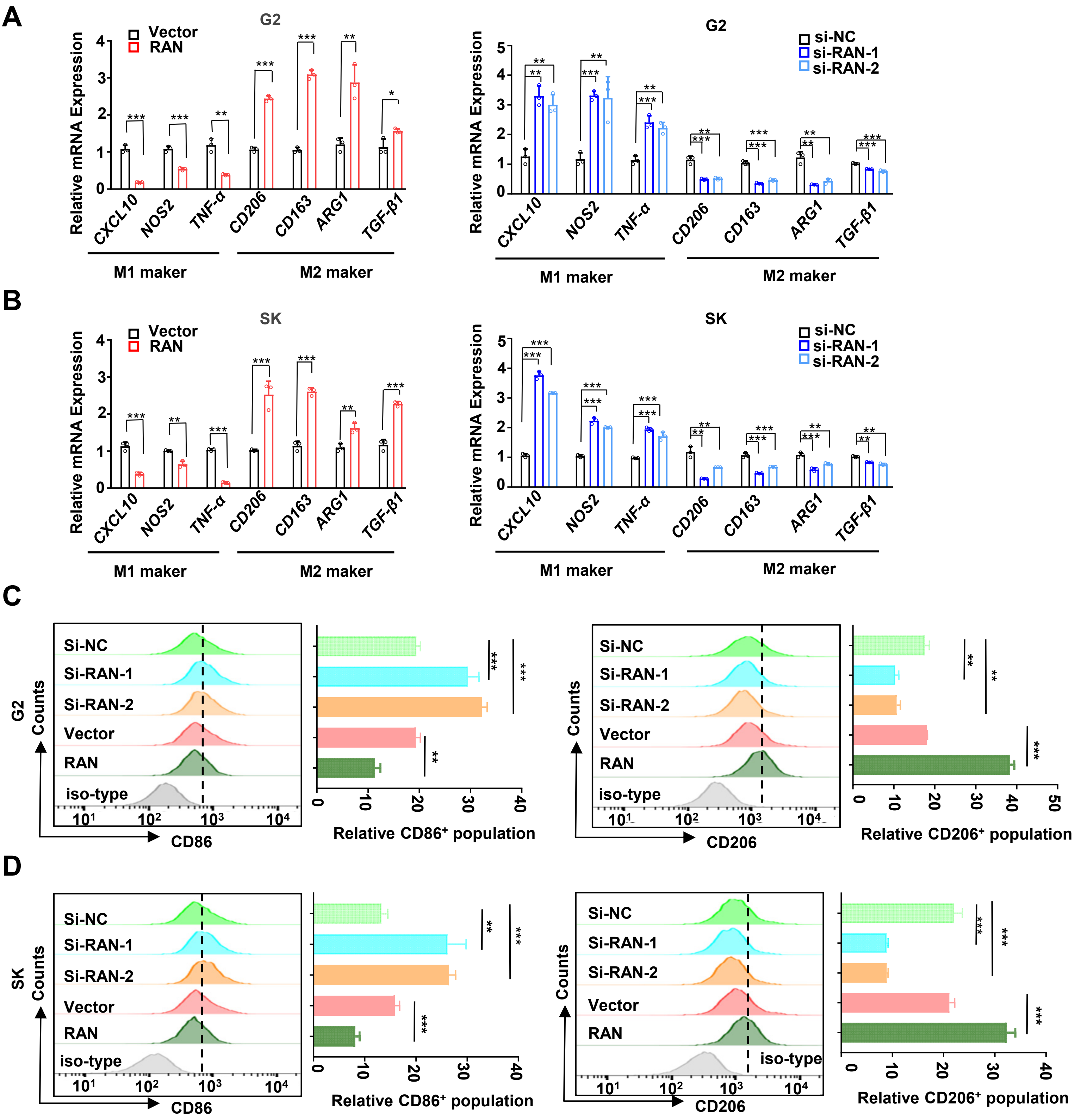

Supplementary Figure 7. RAN enhances M2 polarization of macrophages cocultured with HCC cells.

(A, B) qRT-PCR analyses of M1/M2 macrophage polarization markers in THP-1 cells following co-culture with HepG2 or SK-HEP-1 cells.

(C, D) Flow cytometric analyses of M1/M2 polarization markers (CD86 and CD206) on macrophages following co-culture with HCC cells.

Data information: The difference between two groups was calculated using Student's *t* test. $*P<0.05$, $**P<0.01$, $***P<0.001$.

Supplementary Figure 8

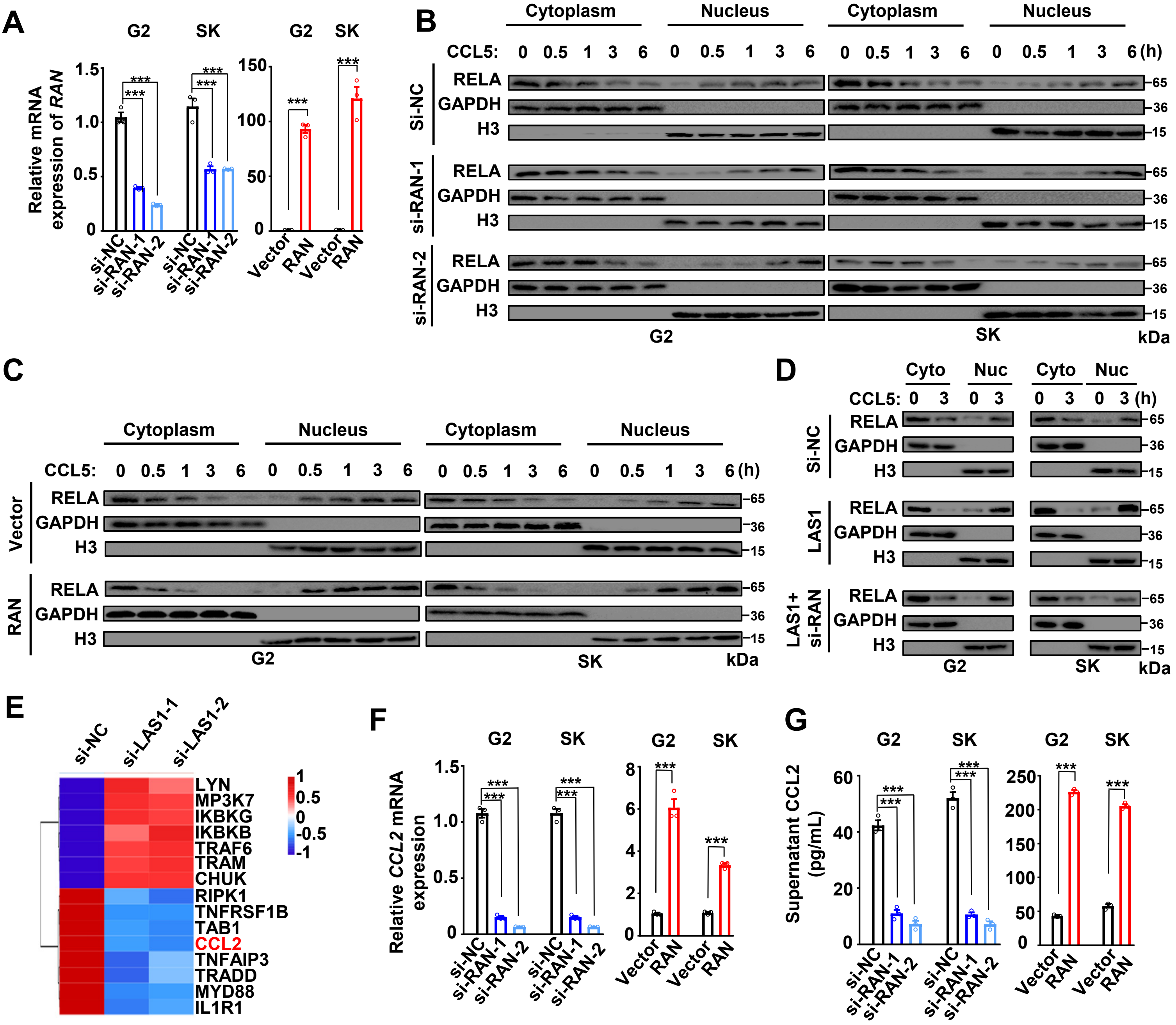

Supplementary Figure 8. *RAN* promotes RELA nuclear import and enhances CCL2 secretion of HCC cells.

(A) The relative expression of *RAN* was examined in HCC cells, where *RAN* was either silenced (using siRNAs) or overexpressed.

(B) The knockdown of *RAN* caused a significant delay in the nuclear entry of RELA in HCC cells induced by CCL5.

(C) The overexpression of *RAN* promoted the nuclear entry of RELA in HCC cells induced by CCL5.

(D) Western blot assays indicated that knockdown of *RAN* inhibited the increased nuclear translocation of RELA due to overexpression of *L1CAM-AS1* in HCC cells.

(E) The heatmap shows the differentially expressed genes in *RAN*-KD cells.

(F) RAN enhanced *CCL2* expression levels in HCC cells.

(G) RAN promoted tumor-derived CCL2 release.

Data information: The difference between two groups was calculated using Student's *t* test. One-way ANOVA analysis with Dunnett's test was used for multiple comparisons. ***$P<0.001$.

Supplementary Figure 9

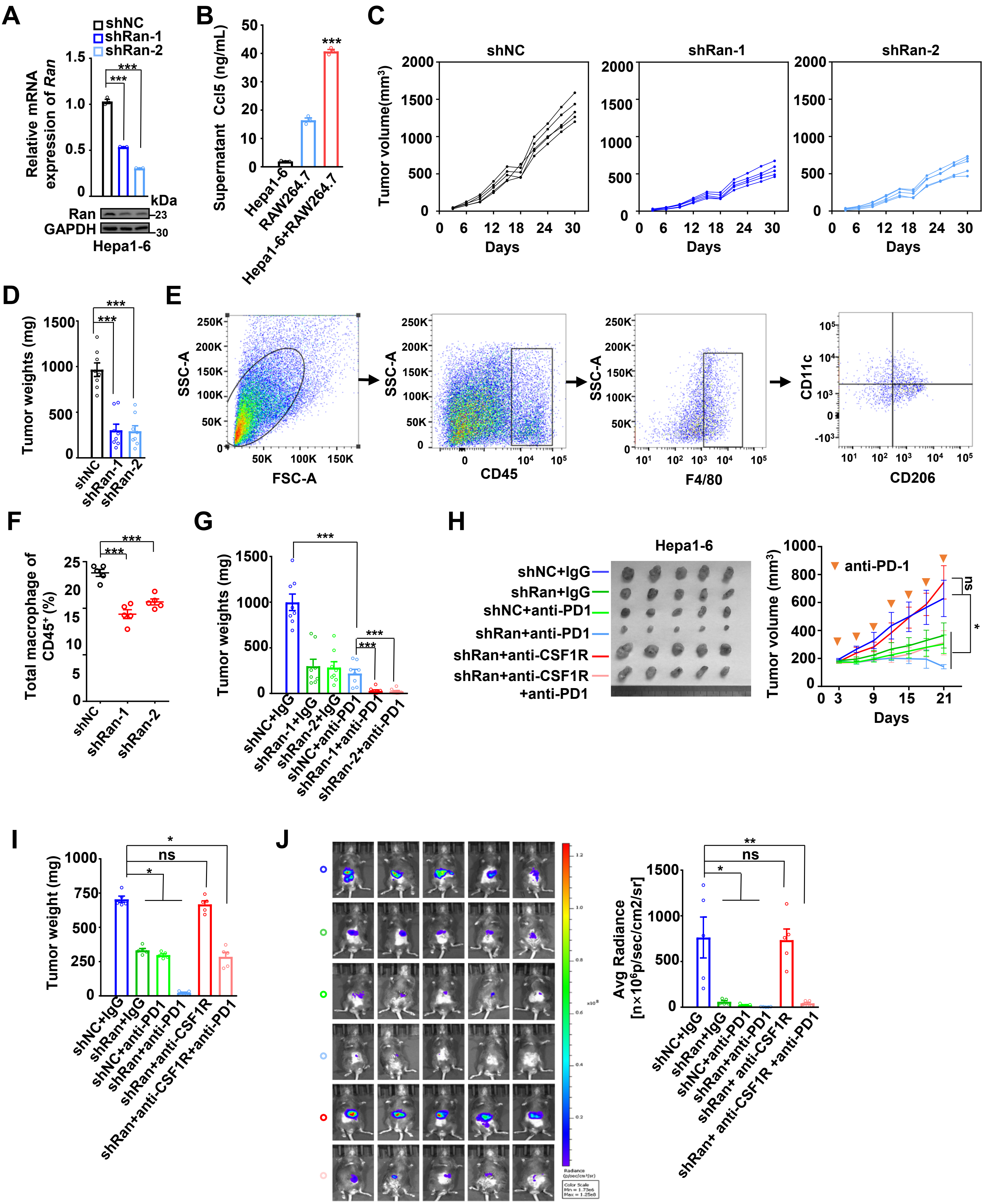

Supplementary Figure 9. Silencing of *Ran* impairs tumor growth and enhances immunotherapy response in tumors.

(A) The relative expression of *Ran* was examined in mouse Hepa1-6 cells where *Ran* was silenced using shRNAs.

(B) The levels of Ccl5 in the supernatants of Hepa1-6, RAW264.7, and co-cultures were quantified.

(C,D) Silencing of *Ran* inhibited proliferation of the Hepa1-6 xenografts.

(E) Gating strategy to identity macrophage subsets.

(F) The *Ran*-KD tumors exhibited evidently reduced total macrophage levels compared to the control xenografts in mice.

(G) Tumor weights of mice with the tumors with or without silencing of *Ran* treated with IgG or the anti-PD1 antibody.

(H) Representative subcutaneous tumor images (left) and growth curves (right) of various groups, including the shRNA+IgG control group (shNC+IgG), the *Ran*-KD+IgG group (shRan+IgG), the shNC+anti-PD1 group (shNC+anti-PD1), the *Ran*-KD+anti-PD1 group (shRan+anti-PD1), the *Ran*-KD+anti-CSF1R group (shRan+anti-CSF1R), or the *Ran*-KD+anti-CSF1R+anti-PD1group (shRan+anti-CSF1R+anti-PD1).

(I) Subcutaneous tumor weights.

(J) Representative pictures (left) and quantitative analyses of bioluminescent signals (right) of the orthotopic HCC model in mice.

Data information: The difference between two groups was calculated using Student's *t* test. One-way ANOVA analysis with Dunnett's test was used for multiple comparisons. *$P<0.05$, **$P<0.01$, ***$P<0.001$, NS, not significant.
