# Supplementary material for: H3K36me3‐Guided m6A Modification of Oncogenic L1CAM‐AS1 Drives Macrophage Polarization and Immunotherapy Resistance in Hepatocellular Carcinoma
Source: Adv Sci (Weinh). 2025 Jun 19;12(33):e14909. doi: 10.1002/advs.202414909 (PMC13001626; doi:10.1002/advs.202414909)
Supplement: Supplementary file 2 — Supporting Information [file ADVS-12-e14909-s002.docx]

Supplementary Table S1. Primers for RT-qPCR

|  | Primer Sequence |
| --- | --- |
| LICAM-AS1-F | GTGCAGCCATTTCGAGCTTT |
| LICAM-AS1-R | CTTCGTGGGGTGTCACTTGT |
| β-actin-F | GGCGGCACCACCATGTACCCT |
| β-actin-R | AGGGGCCGGACTCGTCATACT |
| LICAM-AS1-ChIP-F | CCCACGAAGACAGGGACAAT |
| LICAM-AS1-ChIP-R | ATGGGCAGTCGTGTACTGG |
| L1CAM-AS1-mutF | CATTGCGGGAAAAGGACT |
| L1CAM-AS1-mutR | TTCGCACTGGATACGACTGT |
| METTL3-F | AGAGTGTCGGAGGTGATT |
| METTL3-R | TAGTACGGGTATGTTGAGC |
| METTL14-F | GAGTGTGTTTACGAAAATGGGGT |
| METTL14-R | CCGTCTGTGCTACGCTTCA |
| IGF2BP1-F | GGCCATCGAGAATTGTTGCAG |
| IGF2BP1-R | CCAGGGATCAGGTGAGACTG |
| IGF2BP2-F | AGCTAAGCGGGCATCAGTTTG |
| IGF2BP2-R | CCGCAGCGGGAAATCAATCT |
| IGF2BP3-F | TATATCGGAAACCTCAGCGAGA. |
| IGF2BP3-R | GGACCGAGTGCTCAACTTCT |
| S14-F | GGCAGACCGAGATGAATCCTC |
| S14-R | CAGGTCCAGGGGTCTTGGTCC |
| U2-F | CATCGCTTCTCGGCCTTTTG |
| U2-R | TGGAGGTACTGCAATACCAGG |
| FBXO32-F | TGTGGGTGTATCGGATGGAG |
| FBXO32-R | GAGTTTCTTCCACAGCAGCC |
| OSTM1-F | AGTGTAGCCCAGAGTCTAGTT |
| OSTM1-R | AGGGAGCATATCCCAGAAACA |
| CCL2-F | CAGCCACCTTCATTCCCCAA |
| CCL2-R | GGACACTTGCTGCTGGTGAT |
| c-MYC-F | GTCACACCCTTCTCCCTTCG |
| c-MYC-R | GAGAAGCCGCTCCACATACA |
| Cyclin D1-F | CAATGACCCCGCACGATTTC |
| Cyclin D1-R | CATGGAGGGCGGATTGGAA |
| NOS2-F  NOS2-R | GTCAGAGTCACCATCCTCTTTG  GCAGCTCAGCCTGTACTTATC |
| CXCL10-F  CXCL10-R | AGCTCTACTGAGGTGCTATGT  GTACCCTTGGAAGATGGGAAAG |
| TNF-α-F  TNF-α-R | GAGGCCAAGCCCTGGTATG  CGGGCCGATTGATCTCAGC |
| CD206-F  CD206-R  CD163-F  CD163-R | GGACGTGGCTGTGGATAAAT  ACCCAGAAGACGCATGTAAAG  GGGATGTCCAACTGCTATCAA  GACTCATTCCCACGACAAGAA |
| ARG1-F  ARG1-R | CCCTTTGCTGACATCCCTAAT  GGCTGATTCTTCCGTTCTTCT |
| TGF-β1-F  TGF-β1-R | CCACCTGCAAGACCATCGAC  CTGGCGAGCCTTAGTTTGGAC |
| RELA-F | GTGGGGACTACGACCTGAATG |
| RELA-R | GGGGCACGATTGTCAAAGATG |
| SETD2-F | TGCTTCTAGTCGATTTTTGCCC |
| SETD2-R | AGGGTTTGGAGTATCACTTTGC |
| RAN-F | CATCCCCTAGTGTTCCACACC |
| RAN-R | CATCCCCTAGTGTTCCACACC |
| Mrc1-F | CTCTGTTCAGCTATTGGACGC |
| Mrc1-R | CGGAATTTCTGGGATTCAGCTTC |
| Cd163-F | TCACGGCACTCTTGGTTTGT |
| Cd163-R | ATGACCCCCGAGGATTTCAG |
| Nos2-F | GGAGTGACGGCAAACATGACT |
| Nos2-R | TCGATGCACAACTGGGTGAAC |
| Cxcl10-F | TGAGAGACATCCCGAGCCAA |
| Cxcl10-R | GAGGCAGAAAATGACGGCAG |
| Tnf-α-F | CAGGCGGTGCCTATGTCTC |
| Tnf-α-R | CGATCACCCCGAAGTTCAGTAG |
| Actin-F | GGCTGTATTCCCCTCCATCG |
| Actin-R | CCAGTTGGTAACAATGCCATGT |
| Ran-F | GCGCGGAGACTCTTCTGG |
| Ran-R | TCACGAAGGTTGTCTTCCCG |

Supplementary Table S2. Antibodies used in the study

| Name | Company | Catalog Number |
| --- | --- | --- |
| anti-N6-methyladenosine | Synaptic  Systems | 202003 |
| H3K36me3 | Abcam | ab9050 |
| IgG | Invitrogen | 02-6102 |
| SETD2 | Proteintech | 55377-1-AP |
| METTL3 | Abcam | ab195352 |
| METTL14 | Proteintech | 26158-1-AP |
| IGF2BP1 | Proteintech | 22803-1-AP |
| IGF2BP2 | Proteintech | 11601-1-AP |
| IGF2BP3 | Proteintech | 14642-1-AP |
| GAPDH | Proteintech | 10494-1-AP |
| RAN(Rabbit) | Proteintech | 10469-1-AP |
| RAN(Mouse) | Proteintech | 67500-1-Ig |
| SFN | Proteintech | 10622-1-AP |
| SUPT6H | Proteintech | 23073-1-AP |
| SMARCC1 | Proteintech | 17722-1-AP |
| PTBP1 | Proteintech | 67462-1-Ig |
| RELA | Proteintech | 10745-1-AP |
| FBXO32 | Proteintech | 12263-1-AP |
| OSTM1 | Santa Cruz | sc-293366 |
| Anti-HA  Anti-Flag | Abcam  Sigma | ab9110  F7425 |
| c-Myc | Proteintech | 10828-1-AP |
| Cyclin D1 | Proteintech | 60186-1-Ig |
| IκBα | Proteintech | 10268-1-AP |
| p-IκBα | Proteintech | 82349-1-RR |
| CoraLite488-conjugated Goat Anti-Mouse IgG(H+L) | Proteintech | SA00013-1 |
| CoraLite488-conjugated Goat Anti-Rabbit IgG(H+L) | Proteintech | SA00013-2 |
| CoraLite594-conjugated Goat Anti-Rabbit IgG(H+L) | Proteintech | SA00013-4 |
| PE anti-human CD206 (MMR) Antibody | BioLegend | 321105 |
| FITC anti-human CD86 Antibody | BioLegend | 374203 |
| PE Mouse IgG1, κ Isotype Ctrl Antibody | BioLegend | 981804 |
| FITC Mouse IgG1, κ Isotype Ctrl Antibody | BioLegend | 981802 |
| FITC anti-mouse CD11c Antibody | BioLegend | 117305 |
| PE/Cyanine7 anti-mouse CD45 Antibody | BioLegend | 103113 |
| APC anti-mouse F4/80 Antibody | BioLegend | 123116 |
| PE anti-mouse CD206 (MMR) Antibody | BioLegend | 141706 |
| PE/Cyanine7 Rat IgG2b, κ Isotype Ctrl Antibody | BioLegend | 400617 |
| FITC Armenian Hamster IgG Isotype Ctrl Antibody | BioLegend | 400905 |
| APC Rat IgG2a, κ Isotype Ctrl Antibody | BioLegend | 400511 |
| PE Rat IgG2a, κ Isotype Ctrl Antibody | BioLegend | 400507 |

Supplementary Table S3. Sequences of sgRNAs, siRNAs and shRNAs

| Name | siRNA or shRNA sequence |
| --- | --- |
| sgRNA1-H3K36me3 | Sense 5'-CACCGGGGAGAACCTGGGGTAGTGG-3' |
|  | Antisense 5'-CCCCTCTTGGACCCCATCACCCAAA-3' |
| sgRNA2-H3K36me3 | Sense 5'-CACCGGTCCTGTGAGGAGGTCTGCC-3' |
|  | Antisense 5'-CCAGGACACTCCTCCAGACGGCAAA-3' |
| sgRNA3-H3K36me3 | Sense 5'-CACCGGCATTGTCCCTGTCTTCGTG-3' |
|  | Antisense 5'-CCGTAACAGGGACAGAAGCACCAAA-3' |
| sgRNA4-H3K36me3 | Sense 5'-CACCGGGGTCCAGATACCCTGTCGG-3' |
|  | Antisense 5'-CCCCAGGTCTATGGGACAGCCCAAA-3' |
| sgRNA1-dcas13b | Sense 5'-ATCTGAAGGGAAAAGGGAGGCAA-3' |
|  | Antisense 5'-TAGACTTCCCTTTTCCCTCCGTT-3' |
| sgRNA2-dcas13b | Sense 5'-GTGCCCAGATTGTGCCCCCAATC-3' |
|  | Antisense 5'-CACGGGTCTAACACGGGGGTTAG-3' |
| NC | Sense 5'-UUCUCCGAACGUGUCACGUTT-3' |
|  | Antisense 5'-ACGUGACACGUUCGGAGAATT-3' |
| siSETD2-1 | Sense 5'- GCUCCAUCAAAUCGAUUAATT-3' |
|  | Antisense 5'-UUAAUCGAUUUGAUGGAGCTT-3' |
| siSETD2-2 | Sense 5'-GGUGUAACUUAUGCAUUAATT-3' |
|  | Antisense 5'-UUAAUGCAUAAGUUACACCTT-3' |
| siMETTL3-1 | Sense 5'-GGAGAUCCUAGAGCUAUUATT-3' |
|  | Antisense 5'-UAAUAGCUCUAGGAUCUCCTT-3' |
| siMETTL3-2 | Sense 5'- CUGCACUUCAGACGAAUUATT-3' |
|  | Antisense 5'- UAAUUCGUCUGAAGUGCAGTT-3' |
| siMETTL14-1 | Sense 5'-CCUCCUCCCAAAUCUAAAUTT-3' |
|  | Antisense 5'-AUUUAGAUUUGGGAGGAGGTT-3' |
| siMETTL14-2 | Sense 5'-GGCUAAAGGAUGAGUUAAUTT-3' |
|  | Antisense 5'-AUUAACUCAUCCUUUAGCCTT-3' |
| siIGF2BP1-1 | Sense 5'-GGCCAGUUCUUGGUCAAAUTT-3' |
|  | Antisense 5'- UACAAAGUUAUUAUGGGCCTT-3' |
| siIGF2BP1-2 | Sense 5'- GGCCCAUAAUAACUUUGUATT-3'  Antisense 5'-AUUUGACCAAGAACUGGCCTT-3' |
| siIGF2BP2-1 | Sense 5'-GCCGCAUGAUUCUUGAAAUTT-3' |
|  | Antisense 5'-AUUUCAAGAAUCAUGCGGCTT-3' |
| siIGF2BP2-2 | Sense 5'-CCCGCAUCAUCACUCUUAUTT-3' |
|  | Antisense 5'-AUAAGAGUGAUGAUGCGGGTT-3' |
| siIGF2BP3-1 | Sense 5'-GGCUCAGGGAAGAAUUUAUTT-3' |
|  | Antisense 5'-AUAAAUUCUUCCCUGAGCCTT-3' |
| siIGF2BP3-2 | Sense 5'-GCUGCUGAGAAGUCGAUUATT-3' |
|  | Antisense 5'-UAAUCGACUUCUCAGCAGCTT-3' |
| siRELA-1 | Sense 5'-GCACCAUCAACUAUGAUGATT -3' |
|  | Antisense 5'-UCAUCAUAGUUGAUGGUGCTT -3' |
| siRELA-2 | Sense 5'-GGAGUACCCUGAGGCUAUATT-3' |
|  | Antisense 5'-UAUAGCCUCAGGGUACUCCTT -3' |
| siFBXO32-1 | Sense 5'-GUCGGGAACAUUAACAUGUTT-3' |
|  | Antisense 5'-ACAUGUUAAUGUUCCCGACTT-3' |
| siFBXO32-2 | Sense 5'-GGUACUGAAAGUCCUUGAATT-3' |
|  | Antisense 5'UUCAAGGACUUUCAGUACCTT-3' |
| siOSTM1-1 | Sense 5'-GGAGGCAAAUUGUGCAAAUTT-3' |
|  | Antisense 5'-AUUUGCACAAUUUGCCUCCTT-3' |
| siOSTM1-2 | Sense 5'-GAACCUGGAACACAUUUAUTT-3' |
|  | Antisense 5'-AUAAAUGUGUUCCAGGUUCTT-3' |
| siRAN-1 | Sense 5'-GUGCCAUCAUAAUGUUUGATT-3' |
|  | Antisense 5'-UCAAACAUUAUGAUGGCACTT-3' |
| siRAN-2 | Sense 5'-GGAGACCCUAACUUGGAAUTT-3' |
|  | Antisense 5'-AUUCCAAGUUAGGGUCUCCTT-3' |
| shNC | 5'-CCGGTAGTCGCATACGGAACATTCGCTCGAGCGAATGT  TCCGTATGCGACTATTTTTT-3' |
| shLAS1-1 | 5'-CGGCCCTTCAGATAATCATCAACTCGAGTTGATGATTA  TCTGAAGGGTTTTT -3' |
| shLAS1-2 | 5'-CGGGCCATTTCGAGCTTTGAAACTCGAGTTTCAAAGC  TCGAAATGGCTTTTT -3' |
| shRan-1 | 5'CGGGCGAGTTTGAGAAGAAGTACTCGAGTACTTCTTCTCAAACTCGCTTTTT-3' |
| shRan-2 | 5'CGGGACCCATCAAGTTCAACGTCTCGAGACGTTGAACTTGATGGGTCTTTTT-3' |

Supplementary Table S4. Mass spectrometry of proteins pulled-down by lncRNA L1CAM-AS1 in HepG2 cells

| No. | Gene names | Unique peptides | LFQ intensity Sense | LFQ intensity Antisense |
| --- | --- | --- | --- | --- |
| 1 | SUPT6 | 5 | 1071800000 | 0 |
| 2 | SFN | 5 | 607120000 | 0 |
| 3 | RAN | 4 | 305970000 | 0 |
| 4 | SMARCC1 | 4 | 304320000 | 0 |
| 5 | PTBP1 | 3 | 189950000 | 0 |
| 6 | MYL6 | 3 | 162700000 | 0 |
| 7 | WDR63 | 3 | 122360000 | 0 |
| 8 | ETNPPL | 3 | 97229000 | 0 |
| 9 | FABP5 | 3 | 94833000 | 0 |
| 10 | APRT | 3 | 90081000 | 0 |

Supplementary Table S5. Mass spectrometry of two E3 ubiquitin ligase proteins immunoprecipitated by RAN in HepG2 cells

| No. | Gene names | Unique peptides | LFQ intensity RAN | LFQ intensity IgG |
| --- | --- | --- | --- | --- |
| 1 | OSTM1 | 2 | 631710000 | 0 |
| 2 | FBXO32 | 2 | 89887000 | 0 |
